# Nanomaterials-Mediated Immunomodulation for Cancer Therapeutics

**DOI:** 10.3389/fchem.2021.629635

**Published:** 2021-02-23

**Authors:** Ajita Jindal, Sounik Sarkar, Aftab Alam

**Affiliations:** ^1^ School of Biotechnology, Jawaharlal Nehru University, New Delhi, India; ^2^ Flowcytometry Facility, Modern Biology Department, University of Calcutta, Kolkata, India; ^3^ Department of Clinical Neurosciences, University of Cambridge, Cambridge, United Kingdom; ^4^ Clare Hall, University of Cambridge, Cambridge, United Kingdom; ^5^ Charles River Laboratories, Cambridge Biomedical Campus, Cambridge, United Kingdom

**Keywords:** cancer, nanomaterials, immunomodulation, therapeutics, immunotherapy

## Abstract

Immunotherapy holds great promise in overcoming the limitations of conventional regimens for cancer therapeutics. There is growing interest among researchers and clinicians to develop novel immune-strategies for cancer diagnosis and treatment with better specificity and lesser adversity. Immunomodulation-based cancer therapies are rapidly emerging as an alternative approach that employs the host’s own defense mechanisms to recognize and selectively eliminate cancerous cells. Recent advances in nanotechnology have pioneered a revolution in the field of cancer therapy. Several nanomaterials (NMs) have been utilized to surmount the challenges of conventional anti-cancer treatments like cytotoxic chemotherapy, radiation, and surgery. NMs offer a plethora of exceptional features such as a large surface area to volume ratio, effective loading, and controlled release of active drugs, tunable dimensions, and high stability. Moreover, they also possess the inherent property of interacting with living cells and altering the immune responses. However, the interaction between NMs and the immune system can give rise to unanticipated adverse reactions such as inflammation, necrosis, and hypersensitivity. Therefore, to ensure a successful and safe clinical application of immunomodulatory nanomaterials, it is imperative to acquire in-depth knowledge and a clear understanding of the complex nature of the interactions between NMs and the immune system. This review is aimed at providing an overview of the recent developments, achievements, and challenges in the application of immunomodulatory nanomaterials (iNMs) for cancer therapeutics with a focus on elucidating the mechanisms involved in the interplay between NMs and the host’s immune system.

## Introduction

The emergence of cancer and its advancement are manifestations of the inadequacy of the host's immune system to recognize and execute an effective response to the tumor antigens. The cells of the host’s immune system are responsible for distinguishing and eliminating the cancer cells during the immune surveillance process (Houghton, 1994; de Visser et al., 2006; Lakshmi Narendra et al., 2013). Tumor cells escape the immunosurveillance by evading the anti-tumor measures of the immune system (Liu and Cao, 2016; Muenst et al., 2016). Tumor-derived signals within the tumor microenvironment (TME) downregulates the anti-cancer functions of the infiltrating immune cells. Moreover, they are modulated to promote tumor growth and progression (Whiteside, 2008; Lei et al., 2020). Thus, the immune system plays a paradoxical role by creating an immunosuppressive microenvironment that facilitates cancer progression (de Visser et al., 2006; Kitamura et al., 2015). Immunosuppression can be defined as the diminished ability of the body to induce an immune response due to a lack of immune cells such as T and/or B lymphocytes (Lakshmi Narendra et al., 2013) (For definitions refer Glossary). Chemotherapy is a common treatment for several types of cancers; however, this form of therapy often leads to immunosuppression due to a decrease in the population of immune cells such as T and B lymphocytes (Muenst et al., 2016), and dendritic cells (DCs) (Ferrari et al., 2003; Wertel et al., 2007). However, several chemotherapeutic agents are reported to weaken the immune system by inhibiting the production of blood cells at the bone marrow, resulting in a drastic decrease in the total cell count and impaired immunity (Peng et al., 2015; Liu and Cao, 2016; Muenst et al., 2016). Traditional strategies to combat cancer include cytotoxic chemotherapy, radiation therapy, and surgical interventions. Besides having several adverse side-effects, these approaches also lack the cardinal ability to specifically target the cancer cells while causing minimum damage to healthy cells (Chircop and Speidel, 2014; Pearce et al., 2017). Deleterious side-effects, immunosuppression, multi-drug resistance, and lack of specificity of the existing treatments have necessitated exploration into alternative therapies for cancer. Recently, there has been a paradigm shift in cancer therapeutics towards alternate immunotherapies (Kazemi et al., 2016).

Immunotherapy involves enhancing or suppressing the host’s immune system for the treatment of disease without the utilization of cytotoxic drugs. When applied for cancer treatment, the goal of cancer immunotherapy is to harness the inherent ability of the host’s immune system to eradicate cancer cells in a minimally invasive manner without causing serious side effects, unlike its conventional counterparts (Restifo et al., 2012). There is growing interest among researchers and clinicians aimed at developing novel strategies with better specificity in targeting cancer cells while instigating the least amount of adverse reactions. Recently, some of the alternate approaches to treat cancer are based on immunomodulation which employs the host's own natural defense mechanisms to recognize and selectively eliminate the cancer cells by inducing the immune system (Liu, Z. et al., 2018; Tan et al., 2017; Zhang et al., 2016). Immunomodulation is the process of altering the immune system and its responses. This readjustment of the immune system can either occur naturally, as a part of homeostatic regulation or it can be induced by medical intervention to achieve a therapeutic effect. Besides, this regulation process can be assistive or suppressive in nature to either amplify or abate the immune responses, respectively. In recent years, the technique of induced immunomodulation has gained popularity as a tool for cancer therapeutics.

Nanomaterials can be defined as a class of materials that possess at least one dimension within the range of 1–100 nm (Bleeker et al., 2013). Advances in the field of nanotechnology and material sciences have opened new avenues in the synthesis and development of various nanomaterials (NMs composed of DNA, organic/inorganic polymers, carbon, metals, liposomes, etc. for cancer therapy. The nanostructures can be fabricated in various shapes, sizes, and along with versatile surface functionalities. NMs, when introduced *in vivo*, are recognized by the immune cells as foreign entities therefore generate an immune response that can either be immunostimulatory or immunosuppressive (Yoshioka et al., 2017; Feng et al., 2019). While for most of the applications, these effects are undesirable, but in the case of cancer immunotherapy, the unique immunomodulatory action of NMs can be exploited to target cancer cells.

Recent advances in the field of nanomaterial fabrication and their functionalization has led to the emergence of a new class of multifunctional nanotherapeutic materials that have the potential to revolutionize the field of cancer therapy (Roy Chowdhury et al., 2016). These nanotherapeutic agents exploit several unique properties of NMs such as their ability to specifically target cancer cells, localize at the tumor site, stay in circulation for a longer time period by avoiding clearance, encapsulate and co-deliver multiple active agents, the stimuli-responsive release of cargo, et cetera (Baetke et al., 2015; Chen et al., 2017). Moreover, NM based cancer therapeutic strategies hold great promise for application of personalized medicine as targeted cancer treatments (Diou et al., 2012; Jo et al., 2016).

Interestingly, recent breakthroughs in cancer therapeutics have been for NMs such as gold nanostructures, carbon dots, and carbon nanotubes which possess the ability to cross the blood-brain barrier (BBB). This unique property of NMs creates an opportunity to deliver therapeutic and imaging agents across the BBB and has stimulated widespread research in exploiting their application in brain-cancer treatment and diagnostics (Tang et al., 2019). Additionally, attractive properties of nanoparticles such as a large surface area to volume ratio, tunable dimensions, and, high stability have also imparted beneficial features to the cancer therapeutic regimens. The ability of porous and hollow nanoparticles to load hydrophobic cancer drugs as cargo, protect them from degradation and delivery at the target site in a controlled manner, have revolutionized the designing of cancer nanomedicines (Qin et al., 2017). NMs offer a dual aspect of immunomodulation therapies. They can themselves act as immunomodulatory agents by interacting with the immune system after administration or they can merely act as delivery platforms for targeted delivery of other immunomodulating agents (Kubackova et al., 2020).

**FIGURE 1:**
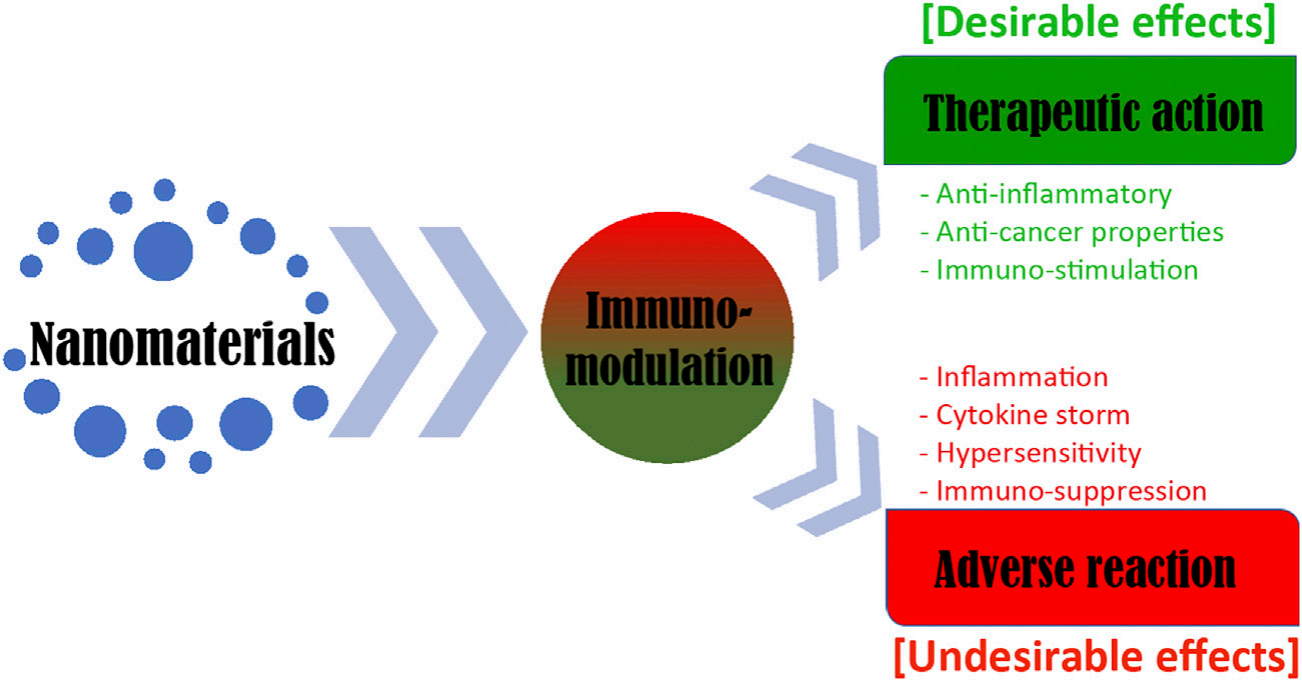
Nanomaterial-mediated immunomodulation for cancer therapeutics.

The immunomodulatory action of NMs can have positive as well as negative impacts (Figure 1). Therefore, the need of the moment is to acquire in-depth knowledge and a clear understanding of the interactions between NMs and the immune system. These insights are vital for the safe application of immunomodulatory nanomaterials (iNMs) for cancer therapeutic applications. In this review, we have focused on the immunomodulatory effects of various NMs and their application towards cancer therapy. The article highlights the role of the immune system in cancer and the interaction of NMs with the immune system to target and eliminate cancer cells either actively or passively. The recent advances in iNMs and their application in cancer therapy are also discussed with a focus on elucidating their mode of action to achieve a better understanding of the nanomaterial-immune system interactions.

## Immune System and Cancer

### The Immune System

Immunity can be defined as the inherent ability of an organism to avert pathogens and diseases by recognition and subsequent neutralization or elimination of foreign entities by the organism’s immune system. These protective mechanisms involve various organs, tissues, cells, and chemical mediators of the immune system and are largely based on the ability to differentiate between the “*self*” and “*non-self*”. The innate (natural) and the adaptive (acquired) immunity are the two major components of the immune system that orchestrate the overall immune responses against foreign pathogens and transformed cells. Therefore, functions of the immune system along with the immune cells acts as a defense barrier against a plethora of invading pathogens as well as cancer in order to restore and maintain homeostasis (Parkin and Cohen, 2001).

Innate immunity consists of pre-deployed molecular and cellular barriers that act against the pathogen within hours of initial contact and therefore serve as the first line of defense of the immune system. Physical barriers include skin, mucosal linings, hair, cilia, etc. that serve as the first line of defense of the immune system. When these physical barriers are breached, the protective role of the soluble mediators and the effectors of the innate system comes into play. The chemical barriers consist of chemical agents present in the antimicrobial peptides, lysozyme, gastric secretions, glandular secretions, small intestine, and blood. Various cell types such as phagocytes (e.g., macrophages, neutrophils, dendritic (DC) cells), and leukocytes (e.g., natural killer (NK) cells, mast cells, eosinophils, basophils) also participate in the innate immune responses as a second line of defense. Another important innate cellular population is that of keratinocytes which are actively involved in recognizing and responding to the pathogen-associated molecular patterns (PAMPs) present on pathogens and danger molecules such as damaged-associated molecular patterns (DAMPs) expressed by the abnormal host cells (Moser and Leo, 2010; Takeuchi and Akira, 2010). Upon recognition by the pattern recognition receptors (PRRs) and subsequent activation of the keratinocytes, cascades involving the release of various soluble mediators (e.g., cytokines, chemokines, complement proteins, collectins, etc.) and antimicrobial peptides (e.g., histatins, cathelicidins, defensins, etc.) are initiated. Therefore, soluble mediators, antimicrobial peptides, and cellular receptors (e.g. Toll-like receptors) are an integral part of the innate immunity that recognizes and responds to the chemical makeup of the pathogens (Braff and Gallo, 2006; Coates et al., 2018). The barriers and the cells of the innate system are the first to encounter and respond to an antigen. Such responses lack specificity but act as a fast and effective primary response against foreign particles (Turvey and Broide, 2010; Iwasaki and Medzhitov, 2015). Moreover, the innate immune system also plays a crucial role in activating the subsequent adaptive immune responses.

On the contrary, the adaptive immune responses are not immediate; they take several days to develop but are highly specific in neutralizing the antigen. Such responses serve as the third line of defense and are acquired by the host’s immune system after encountering an antigen. The T and B lymphocytes orchestrate the two key features of the host's adaptive immune responses; 1) The vast and specific range of T and B cell receptors generated via the process of somatic recombination, and; 2) The ability to develop immunological “memory” upon encountering pathogens leading to a more rapid and potent immune response in case of subsequent encounters (Bonilla and Oettgen, 2010). The cell-mediated adaptive immunity involves cytotoxic T cells that can recognize affected cells and induce cell death (Wing and Remington, 1977). The humoral adaptive immunity is established by helper T cells via activation of B cells which produce specific antibodies against the pathogen. The barriers of adaptive immunity include lymph nodes, lymphoid tissues, and the spleen. The random generation of a wide range of antigen receptors via somatic recombination confers the adaptive immune system with the diverse ability to recognize a plethora of pathogens. However, this diversity comes at a price and can often lead to ineffectiveness in distinguishing between the self and non-self and subsequent attack on the host’s own cells giving rise to autoimmune diseases. Consequently, the immune system has several mechanisms and checkpoints in place for recognition, regulation, and elimination of self-reactive immune cells. Another unique feature of adaptive immunity is the ability to generate custom-tailored antigen-specific T and B lymphocytes upon initial encounter with the pathogen. This ensures a potent and highly specific immune response that is effective in neutralization or elimination of the pathogen. Unfortunately, the process takes a significant period of time (5–6 days) leading to a considerable delay in the elicitation of the adaptive immune responses (Tomar and De, 2014).

In the context of the evolutionary timeline, innate immunity was developed earlier and therefore is found in all multicellular living beings. Whereas, adaptive immunity was an outcome of subsequent evolutionary processes and thus is present only in higher vertebrates (Pancer and Cooper, 2006). Therefore, rather than clear distinct systems, there exists an intertwined network of regulatory mechanisms between the two systems and the overall host defense is a result of a highly complex crosstalk and coordination between the innate and adaptive immunity (Cooper, 2016; Symowski and Voehringer, 2017). Certain cells, proteins, and receptors such as NK cells (Fehniger et al., 2003; Sun and Lanier, 2009), DCs (Palucka and Banchereau, 1999; Chan et al., 2006), cytokines (Belardelli and Ferrantini, 2002; Schön and Erpenbeck, 2018), Toll-like Receptors (TLRs) (Pasare and Medzhitov, 2004), platelets (Elzey et al., 2003; Elzey et al., 2005), are involved in this crosstalk by acting as crucial links between the innate and adaptive immune systems.

Besides acting as a defense barrier against pathogens and eliminating/neutralizing foreign antigens, the immune system also plays an important role in maintaining tolerance towards self and clearing abnormal or damaged host cells that can lead to cancer and various autoimmune disorders (Knochelmann et al., 2018). A diseased condition arises when the immune system is either 1) weak or inactive (immunodeficiency); 2) overactive (hypersensitivity); 3) loses the inherent capacity to distinguish between self and non-self which results in an immune attack towards the host itself (autoimmunity); and/or 4) the antigen/pathogen evades all the existing immune defenses of the host’s immune system leading to the manifestation of the disease (infection) (Kelly and Leonard, 2003; Martinon and Tschopp, 2004; Finlay and McFadden, 2006; Gregersen and Behrens, 2006; Valenta et al., 2009). As the functions of the immune system are impaired in such diseases, positive or negative modulation of the immune responses by activating or suppressing the immune system with the help of immunomodulatory agents serves as an effective mode of treatment. Immuno-suppressive therapies and anti-inflammatory agents are common treatments for diseases related to autoimmunity, inflammation, or hypersensitivities (Ephrem et al., 2005; Broide, 2009; Musette and Bouaziz, 2018; Immunotherapies for Autoimmune Diseases, 2019). Similarly, in the case of immune-deficiencies, upregulation of immune responses via immuno-stimulatory therapies have shown promising outcomes. Immunostimulatory treatments are also effective in treating bacterial and viral infections (Drews, 1980; Dickneite et al., 1984; Smalley Rumfield et al., 2020). Recently, studies are being conducted towards the application of immunomodulation therapies in combating severe acute respiratory syndrome coronavirus 2 (SARS-CoV-2) infection in patients suffering from coronavirus disease 2019 (COVID-19) (Hall et al., 2020; Ingraham et al., 2020; Tay et al., 2020). These therapies are aimed at enhancing the patient’s innate and adaptive immune mechanisms to overcome the pathogenesis of SARS-CoV-2. Additionally, several *in vivo* studies have demonstrated that an impaired immune system leads to higher incidences of clinical manifestations leading to malignant tumors suggesting the importance of immunosurveillance by the immune system in preventing cancer (Shankaran et al., 2001). Therefore, the immune system and its modulation play a critical role in oncogenesis as well as its treatment.

### Role of Immune System in Cancer

Cancer cells can be categorized as altered host cells that evade the growth regulation mechanisms, override the homeostasis maintenance pathways, and undergo uncontrolled growth (Li and Neaves, 2006). The cellular components and biochemical mediators of both innate and adaptive immunity are involved in a complex crosstalk to give rise to an effective anti-tumor response consisting of processes such as immunoediting and the cancer immunity cycle (Swann and Smyth, 2007; Calì et al., 2017). Adaptive immunity is responsible for the generation of antigen-specific immune responses; hence, it plays a central role in eliciting anti-cancer responses against the cancer tumor antigens.

#### Anti-Cancer Measures by the Immune System

The host’s immune system coordinates a highly complex series of sequential steps to eliminate the cancer cells. These events lead to the development of effective anti-cancer responses which altogether constitute the “cancer immunity cycle” (Chen and Mellman, 2013). During the early stages of cancer development, the tumor/cancer immunity is established when tumor antigens are recognized by dendritic cells (DCs) of the host’s immune system and the antigens are processed and presented on the surface of antigen-presenting cells (APCs) via major histocompatibility complex (MHC) I or II. DC cells are a crucial link between innate and adaptive immunity and orchestrate the response to antigen by processing and presenting it to the cells of the adaptive immune system. They are responsible for priming and activating the naïve T cells located within the draining lymph nodes (DLNs) via interaction with MHC complex and co-stimulatory signals. This leads to the maturation of T cells to effector T cells that overexpress certain receptors. Regulatory T cells (Tregs) play a key role in modulation of the immune system to maintain tolerance towards self-antigens. Tregs have immunosuppressive effects as they usually downregulate the functioning of T effector cells. Studies have shown that the depletion of the Tregs population at the tumor site helps to overcome the immunosuppressive TME and significantly improves the efficacy of cancer immunotherapies (Curiel, 2007; Wang et al., 2017). Once activated, the circulating T effector (Te) cells reach the tumor site and are responsible for evoking an immune response against the cancerous cells. The cytotoxic T lymphocytes (CTLs) recognize tumor cells via MHC-I and eliminate them by releasing chemical cues such as granzyme B and perforins. The macrophages and mast cells release soluble chemical mediators such as cytokines, histamine, chemokines, and reactive oxygen species (ROS) when they encounter the tumor antigen. The chemical mediators initiate inflammation and tissue repair. The obliteration of tumor cells, in turn, releases more tumor-derived antigens that lead to the establishment of a more robust cancer-immunity. Therefore, the key players in establishing an effect on tumor immunity are macrophages, DCs, cytotoxic T lymphocytes (CTLs), and regulatory (Tregs) T cells (Chen and Mellman, 2017). Additionally, for specific and effective elimination of cancerous cells, the activity of CTLs and macrophages should be stimulated while the function of Tregs inhibited (Ruter et al., 2009).

#### Recognition and Elimination by the Immune System

Cancer immunoediting comprises of three phases (Dunn et al., 2004). The first is the elimination phase which involves immunosurveillance by the innate and adaptive immune systems to detect and eliminate the abnormal cells that are either malignant or have the potential to become malignant. The elimination phase involves several cell populations such as NK cells, macrophages, DCs, cytotoxic T cells, and B cells (Dunn et al., 2006). This stage marks the undetectable phase of early tumor development. The tumor cells that have escaped the initial stage of elimination enter the next phase of equilibrium where the tumor cells coexist with the host’s immune system. This is the longest phase and marks a temporary state of equilibrium between the host’s immune system and the tumor. Moreover, during this period, the tumor cells can either remain dormant or can accumulate mutations giving rise to new tumor variants that in turn increases the overall resistance of the tumor to the host’s immune mechanisms (Mittal et al., 2014). The growth and expansion of tumor cells may eventually lead to the escape phase where the cancer cells can metastasize and give rise to malignancies (Dunn et al., 2002; Smyth et al., 2006).

#### Escape and Evasion by the Tumor Cells

As the tumor growth progresses, some of the tumor cells may evade the immune system’s measures and escape. Most of the evasion mechanisms of the tumor cells facilitate the development of a TME that can protect the cancer cells by suppressing the host’s immune responses (Schreiber et al., 2011). Therefore, immunoediting is a continuous process involving both immunosurveillance to eliminate cancer cells and the evasion by tumor cells that leads to tumor progression. The escape of tumor cells from various stages of immunoediting is mediated by numerous immunosuppressive strategies. Some of these evading mechanisms include downregulation or absence of MHC class I expression on cancer cells preventing T cell-mediated responses, production of cytokines that induce anti-apoptotic pathways, upregulation of non-classical MHC class I expression preventing NK mediated cell killing and tumor cell-induced immunosuppression (Waldhauer and Steinle, 2008; Al-Tameemi et al., 2012; Mohme et al., 2017). Escape and evasion of tumor cells often leads to cancer malignancies, creating the need for clinical interventions in the form of various cancer therapies.

### Emergence of Cancer Immunotherapies

**FIGURE 2:**
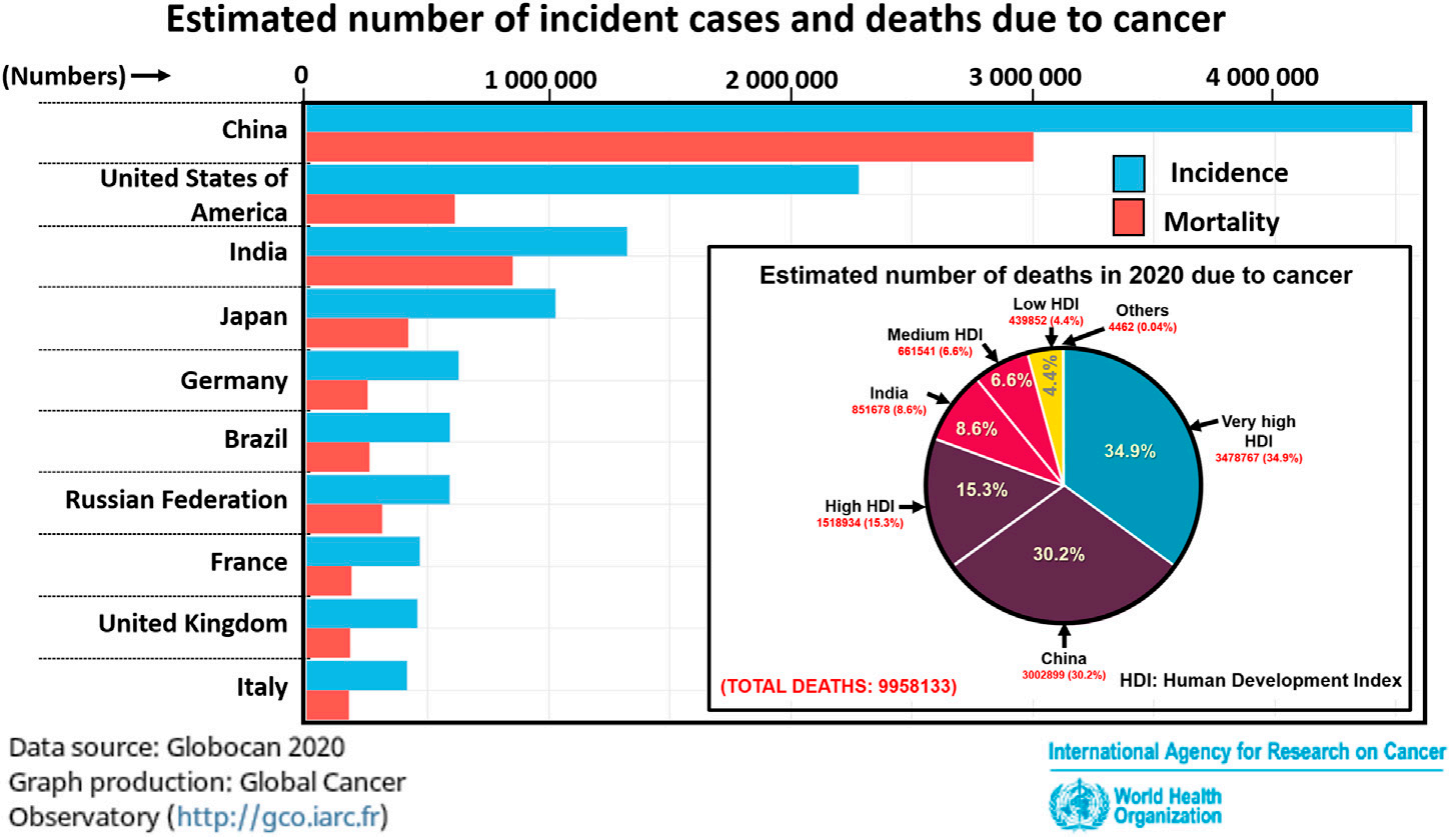
Cases of cancer prevalence and mortality in the year 2020. In inset: Estimation of mortalities due to cancer in 2020 among countries based on human development index (HDI). Statistics provided by Globocan 2020, World Health Organization.

Cancer is one of the major causes of death around the world. Figure 2 depicts the prevalence of cancer and cancer-related mortalities across major countries based on the latest Globocan 2020 report. In the year 2020 alone, cancer was responsible for approximately 9.9 million deaths globally out of which about 65% of the deaths were attributed to under-developed and developing countries (Figure 2 (inset)).

The conventional cancer treatments are based on surgical intervention, radiation therapy, and/or chemotherapy. Even though these procedures are highly invasive and involve deleterious side-effects, they constitute a major part of the standard cancer treatment methodology (Cosentino and Piro, 2018; Pusuluri et al., 2019). These treatments are applied either individually or in combination to combat cancer and its recurrence. However, these conventional methods of treatment are associated with several limitations such as poor solubility due to the hydrophobic nature of the drug, lack of selectivity and specificity, immunosuppression, multidrug resistance, high rate of metastasis and recurrence. These limitations have encouraged the development of alternative therapies based on modulation of the immune system and its responses to better combat cancer either independently or in conjugation with the conventional treatments.

The therapeutic method based on modulating the host’s own immune system to eliminate cancer was first proposed in the early 1890s (Coley, 1991). Unfortunately, the novel technique failed to draw the attention of the oncologists due to the lack of knowledge and understanding of the underlying mechanisms of the immune system. Consequently, over the new few decades, a treatment plan consisting of conventional procedures such as surgery, radiation and/or chemotherapy prevailed as a gold standard for treating cancer.

**FIGURE 3:**
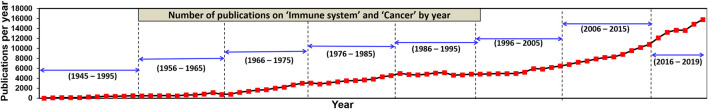
Year-wise number of publications on 'Immune system' and 'Cancer' from the year 1945–2019. As per the data available on PubMed.

**FIGURE 4:**
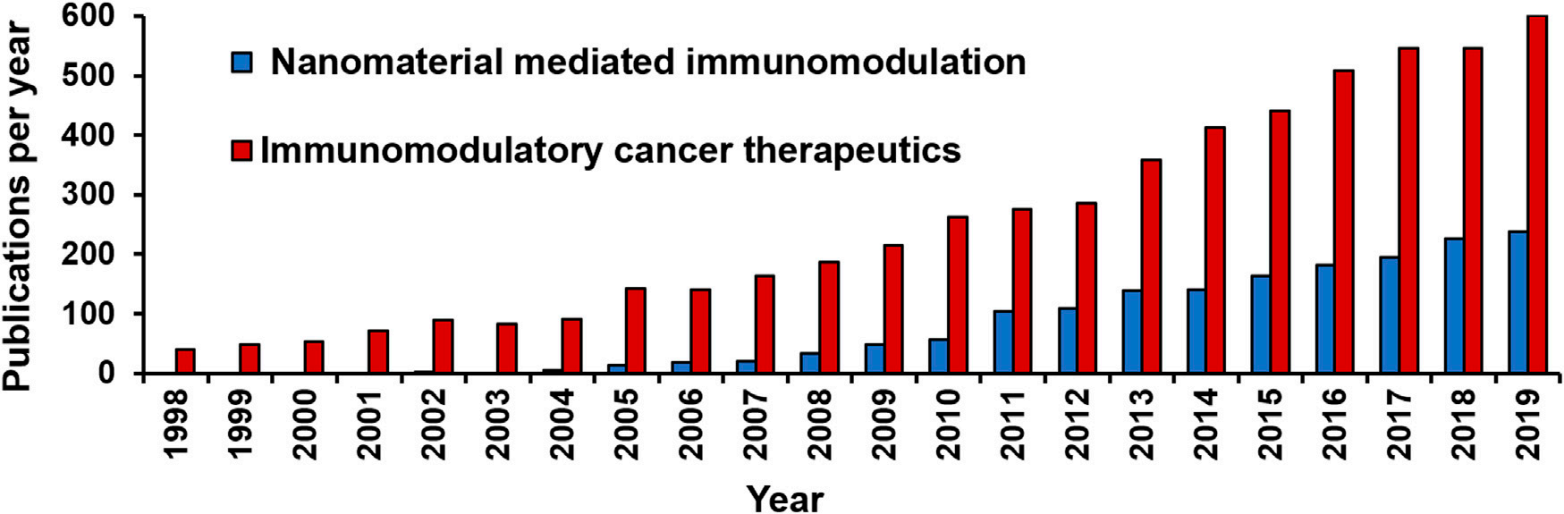
Number of publications on “Nanomaterial mediated immunomodulation” and “Immunomodulatory cancer therapeutics” indexed in PubMed from year 1998–2019.

However, simultaneously the following decades saw a steady rise in the number of studies aimed at unravelling the intrinsic relation between the immune system and cancer (Figures 3, 4). Along the path, a vast knowledge has been gathered by elucidating the role of the host’s immune cells in establishing anti-tumour responses and the evading mechanisms of the cancer cells. The in-depth understanding of the immune mechanisms and the severe limitations of conventional cancer treatments led to the revival of the immunomodulatory approach towards combating cancer (D'Errico et al., 2017; Decker and Safdar 2009).

As a result, in the early 2000s, the concept of immunotherapy was revisited as an alternative approach to treat cancer (Zacharski and Sukhatme, 2005). Unlike the traditional treatments, these therapies are based on boosting the host’s immune responses by administering cytokines, antibodies and/or various co-stimulatory signals. Therefore, they have fewer side-effects and are much more effective in eradicating primary tumours and preventing future incidences of recurrence and metastasis (Kroemer and Zitvogel, 2018). Thus, immunotherapy served as a cornerstone for a paradigm shift in the battle against cancer and has become a standard part of the cancer treatment regimen (Abbott and Ustoyev, 2019). Till today, several cancer immunotherapies have been developed based on stimulation of DCs (e.g. DC-based vaccines) (Sabado et al., 2017; Perez and De Palma, 2019), enhancing the T-cell mediated immune responses (e.g. Adoptive T-cell therapy and chimeric antigen receptor (CAR) T-cell therapy) (Met et al., 2019; Miliotou and Papadopoulou, 2018) and by blocking the inhibitory checkpoints that suppress the immune responses towards cancer (e.g. Checkpoint inhibitor therapy) (Ribas and Wolchok, 2018; Xu, W. et al., 2020). In 2018, the Nobel Prize in Physiology or Medicine was awarded to James P. Allison and Tasuku Honjo for their ground-breaking work on cancer immunotherapy via inhibition of negative regulation (Ballas, 2018). Despite the novel approach of cancer immunotherapy techniques, the extent of their clinical applications has been largely restricted due to significant limitations. For example, cancer patients undergoing checkpoint-inhibition immunotherapies are often prone to developing immune-related adverse events and autoimmune diseases (Dhodapkar, 2019; Pauken et al., 2019). Cancer immunotherapies have shown limited effectiveness in the treatment of solid tumours due to the inability of the immune cells to infiltrate the solid tumour and its microenvironment (Melero et al., 2014). Only a fraction of cancer patients responds to administered immunotherapies and the ones who respond often develop acquired resistance to these therapies (Draghi et al., 2019; Hegde and Chen, 2020). Moreover, certain tumours are characterized as “cold” due to the lack of infiltrating T cells indicating the presence of an immunosuppressive TME that discourages immune activation (Bonaventura et al., 2019). Such “cold” tumours act as a major challenge as they show poor response towards traditional cancer immunotherapies. Thus, much of cancer research has been focused on turning these “cold” tumours into “hot” tumours by modulating the TME and boosting the host’s immune system (Duan et al., 2020).

The technological advances in the field of nanotechnology hold a great promise towards the development of better-targeted cancer immunotherapies with higher specificity, stability, and sustained antitumour effects (Shukla and Steinmetz, 2016). Due to their small size, NMs can effectively infiltrate solid tumours, display retention properties, escape clearance, remain in circulation for longer periods and their surfaces can be easily functionalized to bear specific targeting moieties (Liu, Y. et al., 2018). They can also be applied as nano-delivery systems that can encapsulate various small active agents and deliver them in a sustained manner at the target site (Sarkar et al., 2013; Asadi et al., 2017). When compared to their free forms, the NM encapsulated formulations of several drugs have shown better toxicity profiles with lesser side-effects upon administration thereby significantly improving the therapeutic index of these potent drugs (Desai et al., 2006; Fukuda et al., 2017).

In recent times, several cancer immunotherapies have been aimed at targeting the stages of the cancer immunity cycle. The latest advances in this research area include the application of nanomaterials for formulating improved cancer immunotherapies to achieve enhanced tumor-targeting and neutralizing the immuno-suppressive TME (Yang and Zhang, 2020). A recent work involving the fabrication of a three-in-one nanoplatform was successful in amplifying the various phases of the cancer-immunity cycle to boost the overall immune responses against cancer antigens. The immunotherapy was based on NM-mediated delivery of an immune-agent to elicit a robust immune action along with memory responses. Therefore, it acted as a potent nano-weapon for the prevention of tumor metastasis and future relapses (Li Q. et al., 2019). Another interesting study based on liposomal co-delivery of imatinib (IMT) and siRNA-PD-L1 showed promising results towards enhancement of the cancer immunity cycle by knockdown of programmed cell death ligand 1 (PD-L1) which in turn prevented the PDL1-mediated immunosuppression of tumor-specific CTLs (Li and Han, 2020).

Nanoparticles can infiltrate the “cold” tumours allowing co-delivery of a wide variety of payloads specifically to the tumour site thus boosting the immune activation at TME. Additionally, the immunomodulatory properties of the NMs also aid in turning “cold” tumours into “hot” (Rodallec et al., 2018; Buabeid et al., 2020). For example, a recent study based on citrate-coated superparamagnetic iron oxide NPs (SPIONs) display the capacity to act as a magnetic drug delivery system for guided targeting to “cold” tumours by application of an external magnetic field (Mühlberger et al., 2019). The application of NMs in cancer immunotherapies has opened new arenas to integrate therapy and diagnosis into a single nano-therapeutic unit, leading to a paradigm shift in onco-treatments and paving way for novel developments in the field of cancer therapy and diagnosis (Tan et al., 2020). Another recent interesting study was based on the utilization of a zeolitic imidazole framework (ZIF) as a nanoplatform for pH-responsive release of cargo molecules for targeted combinational therapy to treat “cold” tumours. The nanoplatform was loaded with a photothermal agent (indocyanine green) and an immune adjuvant (imiquimod). The multifunctional nanoplatform eradicated the primary tumours in *in vivo* tumour models and also prevented tumour recurrence when re-challenged (Yu et al., 2020). Since the success of any immunotherapy-based cancer therapy depends largely on enhancing the inherent cancer immunity of the patient, a comprehensive understanding of the underlying mechanisms of immunomodulation is an essential pre-requisite for designing an efficient clinical strategy.

## Immunomodulation as a Tool in Cancer Therapeutics

Immunomodulation is the process of altering the immune system and its responses. Such alteration can be of two types: immuno-suppression–where the immune responses are diminished or immuno-stimulation–where the immune responses are amplified (Grabowski et al., 2020; Nishiguchi and Taguchi, 2020). This fine-tuning of the immune system can either occur naturally, as a part of homeostatic regulation or it can be induced by medical interventions to achieve a therapeutic effect. Immunosuppressive therapies are usually applied in the case of auto-immune diseases (Carballido et al., 2020) and immuno-stimulation is a course of action for cancer and infectious diseases (Dittmer and Olbrich, 2003; García-Martínez et al., 2018). The recent trend is to apply nanotechnology to design alternative treatment approaches which can induce therapeutic effects at the target site while causing minimum collateral damage and adverse effects (Li, T. et al., 2020; Tan et al., 2020). Unlike conventional cancer therapies, this approach provides a more precise and personalized therapeutic effect that causes minimum side-effects and allows simultaneous imaging of the target site (monitoring) (Hapuarachchige and Artemov, 2020; Xu, C. M. et al., 2020). As a result, the technique of induced immunomodulation has gained popularity as a tool for cancer therapy (Comparetti et al., 2018; Li, J. et al., 2019; Xu, B. et al., 2020). Since these immunotherapy-based approaches towards eradicating cancer cells rely on employing the body’s own natural defense system to combat cancer they achieve targeted results with fewer side-effects than conventional cancer treatments (Sau et al., 2019).

NMs have gained a lot of attention in the field of biomedical and therapeutics due to several unique set of features offered by this class of smart materials. These properties of NMs are discussed in detail in the following Properties of Nanomaterials (NMs) Section of this article. Over recent years, several types of NMs have been investigated for their ability to react with components of the immune system and modulate its behavior. The modulation of the immune system via nanomaterials is a complex process and can have both beneficial and harmful effects (Alsaleh and Brown, 2018). Also, the nanomaterial-mediated immunomodulation can be achieved either directly- where the NMs itself causes immunomodulatory effects or indirectly- where the NMs acts as a vehicle for targeted delivery of immunomodulatory agents (IMAs) (Bartneck, 2017; Kubackova et al., 2020). Moreover, due to the small size of NMs and the leaky vasculature of tumors, the NMs undergo enhanced permeability and retention (EPR) effects that allow them to access tumors and selectively co-localize within them (Shi et al., 2020). Therefore, nanomaterial-mediated immunomodulation has the ability to execute targeted delivery of cargo molecules to the immune cells, resulting in better clinical outcomes with minimal adverse events. These unique properties have resulted in a fast-growing interest in the field of NM-mediated immunomodulation and application in cancer therapy during the past decade.

## Mechanism of Immunomodulation by Nanomaterials

### Properties of Nanomaterials

Over the last few decades, advancements in the field of nanotechnology and material sciences have led to a major breakthrough in the fabrication of various NMs composed of DNA (Dai et al., 2017; Jiang et al., 2017), polymers (Chang et al., 2018; Simpson et al., 2019), carbon (Teradal and Jelinek, 2017; Jiang et al., 2019), metals (Dhivya et al., 2015; Singh, 2018), composites (Song et al., 2015; Zhai et al., 2018), liposomes (Zhang et al., 2011; Lorente et al., 2018; Zhang et al., 2018), *et cetera* for cancer therapy. Synthesis of NMs allows the materials to be fine-tuned to tailor their properties (e.g., size, composition, surface properties, etc.) according to the targeted biological application (Wang et al., 2009). The NMs within the dimension range of 1–100 nm closely resemble the natural hierarchy and architecture of human cells, tissues, and ECM at a nanoscale. This confers NMs with the ability to interact with their surroundings, improving the effectiveness, accuracy, and efficacy of the nanomaterial-mediated therapeutic and diagnostic approaches (Li, B. et al., 2018; Tan and Saltzman, 2004). Additionally, attractive properties of NMs such as a large surface area to volume ratio, tunable dimensions, ease of surface functionalization, and, high stability have also imparted beneficial features to the cancer therapeutic regimens (Sun et al., 2014). Various works have also reported conferring “stealth” properties to nanomaterials by attachment of surface molecules such as polyethylene glycol (PEG) (Suk et al., 2016) and CD47 molecules (Gheibi Hayat et al., 2019) to prolong their systemic circulation time by delaying *in vivo* clearance (Fam et al., 2020). A recent study on the fabrication of nano-artificial antigen-presenting cells (aAPCs) decorated with both PEG and CD47 moieties have shown evasion of nanoparticles to phagocytic clearance along with their ability to induce the generation of antigen-specific T cells to suppress tumour development (Song et al., 2019). Another promising feature of NMs towards drug delivery applications is the ability to easily functionalize their surfaces with various ligands to improve cellular uptake and achieve a highly specific targeted drug delivery (Cisterna et al., 2016; Sykes et al., 2016).

Due to the enhanced surface area to volume ratio, the porous and hollow nanomaterials display excellent drug loading efficiencies (Yang et al., 2019). Such NMs are capable of efficiently encapsulating the hydrophobic (water-insoluble) cancer drugs as cargo within their framework, thereby improving the stability of drug molecules, protecting them from degradation, improving their bioavailability, and delivering them to the target site in a controlled manner (Kashi et al., 2012). The delivery of therapeutic agents (TAs) conjugated within nanomaterials also increases their circulation time, allows cellular- and/or tissue-specific targeting, and a stimuli-responsive controlled release of TAs at the target site. These factors drastically reduce the adverse effects of the TAs on the nearby cells and tissues (Iglesias et al., 2018). For instance, in the past decade, mesoporous silica nanoparticles (MSNs) have been exploited as stimuli-responsive nano-carriers for targeted delivery of a wide range of small molecules for diagnostic as well as therapeutic approaches. Major advantages of using MSN-type nano-systems for delivery of cytotoxic cancer drugs are improved drug efficacy, stimuli-responsive controlled release at the target site, and reduced toxic effects to nearby healthy tissues (Alyassin et al., 2020; Aquib et al., 2019; Li, T. et al., 2019; Nguyen et al., 2019). Another area of recent breakthroughs in cancer therapeutics has been in the field of NMs [such as gold nanostructures (Cheng et al., 2014; Lee et al., 2017), carbon dots (Li et al., 2016; Liyanage et al., 2020), carbon nanotubes (You et al., 2019), liposomes (Vieira and Gamarra, 2016; Jena et al., 2020), etc.] that possess the ability to cross the blood-brain barrier (BBB). This unique property of such NMs creates an opportunity to deliver therapeutic and imaging agents across the BBB and has stimulated widespread research in exploiting their application in brain-cancer treatment and diagnostics (Tang et al., 2019). Recently, an *in vivo* study conducted in mice has reported the first successful application of a polymeric nano-immuno-conjugate to deliver immune-therapeutics across BBB and stimulation of local antitumour immune response for the treatment of glioma tumours (Galstyan et al., 2019).

However, the adverse effects associated with the delivery of cytotoxic agents along with the increased chances of drug resistance largely overshadow the therapeutic effects of such an approach (Weber et al., 2015; Babiker et al., 2018; Makovec, 2019). This has led to the emergence of a much efficient treatment regimen for the eradication of tumours involving modifying the body’s own immune responses towards the tumour cells (Irvine et al., 2013). As described in the previous section, such immunomodulatory therapies are aimed at converting immunosuppressive TME into an immunostimulatory one, facilitating the elimination of cancer cells. This also leads to the generation of memory T-cells against cancer antigens thereby discouraging incidences of metastasis and future relapses (Curti et al., 2013; Locy et al., 2018). Consequently, recent works in the area of cancer therapeutics have been majorly focused on utilizing various biomolecules and nanomaterials to increase the specificity and efficacy of direct and/or indirect immunomodulation (Chidambaram et al., 2011). NMs have the potential to be utilized as a delivery system that allows *in vivo* imaging, sustained cargo release, enhanced circulation time, targeted delivery, and stimuli-responsive release of cargo molecules and multiple agents (Li, L. et al., 2018).

### Interaction of NMs With the Immune System

The in-depth knowledge about the components of the immune system and their role in regulating numerous diseases when combined with the recent technological advancement in the field of fabrication of safe and biocompatible NMs have revolutionized the biomedical applications of nanomaterials. Nanomaterial-based therapies have been applied for the treatment of autoimmune diseases (Gao et al., 2017; Kwiatkowski et al., 2020), neurodegenerative diseases (Fornaguera et al., 2015; Turjeman et al., 2015), inflammatory diseases (Li, C. W. et al., 2020; Tran et al., 2015), and cancer (Xin et al., 2017; Samanta et al., 2019). When introduced *in vivo*, NMs act as foreign entities and consequently lead to various immune responses. Nearly all the NMs exert immunomodulatory effects *in vivo* which can be either immunostimulatory or immunosuppressive in nature. The unique ability of NMs to interact with components at a nanoscale and bring about change can lead to both therapeutic as well as deleterious events. As a result, in most of the biomedical applications, the modulatory effects of NMs are undesirable as they often lead to adverse effects. For instance, several recent *in vitro* and *in vivo* studies have demonstrated the severe immunotoxicity associated with the use of single-walled carbon nanotubes (SWCNTs) for biomedical applications (Sayes et al., 2006; Tian et al., 2006; Wang et al., 2011). The surface chemistry, concentration, density of functionalization, and surface area of SWCNTs critically influence their interaction with macrophages and the immune organs such as the spleen (Sayes et al., 2006; Zhang et al., 2011; Lee et al., 2015).

However, at times, the ability to modulate immune responses by NMs have shown compelling results and serves as a tool for immunomodulatory therapies. Therefore, NMs have found a wide-scale application as a tool to mediated immunomodulation either directly by interacting with the components of the immune system or indirectly by acting as a carrier for the delivery of immunomodulatory agents. Recent studies based on NM-mediated direct immunomodulation and NMs as a delivery system for IM agents are listed in Supplementary Tables S1 and S2 (supplied as Supplementary Material), respectively.

## NM Mediated Direct and Indirect Immunomodulation

A variety of immunomodulatory NMs with a unique plethora of physical and chemical attributes have been fabricated for stimulating the immune system to combat cancer. For example, the distinctive ability of the platelets to target cancer cells and their inherent capacity to load drug molecules have been utilized to develop targeted drug delivery nanosystems for anti-cancer therapies. Liposome-based delivery systems are a popular platform for targeted delivery and sustained release of anti-cancer agents (Mukherjee et al., 2019). Various liposomal formulations have been developed for the delivery of immunomodulatory agents (IMAs) that allow modulation of the TME to establish an overall antitumor immune response again tumour antigens (Gilabert-Oriol et al., 2018). Recent studies have also highlighted the inherent tendency of liposomes to interact extensively with the immune system leading to several immunomodulatory effects on the growth of the tumour (La-Beck et al., 2019). Unique core-shell structure allows simultaneous co-delivery of multiple cargo molecules, *in vivo* protection against degradation and the facile surface chemistry can be applied to decorate the NM surface with active targeting moieties, thus making liposomes an excellent candidate for cancer therapeutic applications.

Additionally, several bio-inspired NMs have also gained popularity due to their enhanced biocompatibility and cyto-friendly nature, eco-friendly mode of synthesis, superior bioactivity when compared to the chemically synthesized NMs (Mukherjee and Patra, 2017). Biosynthesized metallic nanoparticles, especially gold (AuNPs) and silver (AgNPs) have been widely studied for achieving targeted cancer therapy and diagnosis (Balakrishnan et al., 2017; Mukherjee et al., 2015; Mukherjee et al., 2016). For example, a study based on biosynthesized colloidal AgNps prepared from leaf extract showed enhanced antibacterial and anti-cancer activity than the chemically synthesized NPs. The multifunctional AgNPs were biocompatible and exhibited bright red fluorescence upon intracellular localization emphasizing their potential to be utilized for biomedical diagnostic tools (Mukherjee et al., 2014). Thus, immunomodulatory NMs possess unique and tunable properties that hold great potential to be utilized as cancer therapeutic agents.

## Clinical Studies and Perspectives

Regardless of the advantages of NMs, very few NM-based immunomodulatory cancer therapies have been actively translated to clinical application majorly due to the limited knowledge about the behavior of NMs when introduced *in vivo* and their long-term toxicity (Sun et al., 2019). The unique properties of NMs, while being attractive for various biomedical applications, at times also raise quite a few biosafety concerns. For example, due to the surface properties such as hydrophobicity and surface charge, NMs when introduced *in vivo* tend to agglomerate into larger particles that might lead to accumulation and toxicity (Pattan and Kaul, 2014). Moreover, as a result of their small size, NMs have the ability to penetrate cells and organelles. Several studies have also shown an increase in intracellular oxidative stress and damage when cells are exposed to NMs such as carbon nanotubes (Francis and Devasena, 2018), gold nanoparticles (Chen et al., 2009), copper nanoparticles (Meng et al., 2007), titanium NMs (Demir, 2020), etc. These events often lead to adverse effects such as DNA aberration, genetic instability and inflammation depending upon the cell type, nature of NM and material concentration (Demir, 2021).

Despite the limitations, some of the NM-based immunomodulatory therapies that are undergoing clinical trials hold immense potential towards a safe and successful clinical application in cancer therapeutics. For example, a recent human trial consisting of six patients suffering from glioblastoma (GBM) involved the use of superparamagnetic iron oxide nanoparticles (SPIONs) via the “NanoPaste” technique. The resection cavities were coated with SPIONs after which the patients were subjected to six sessions of hyperthermia using alternating magnetic field lasting for about 1 h each. No major side-effects were observed during the ongoing treatment. After 2–3 months of treatment, the imaging analysis revealed the formation of NP deposits that displayed signs of inflammation and edema. Further analysis indicated that the treatment was effective in eliciting an antitumor response by tumor necrosis, infiltration of macrophages following uptake of NPs, increasing CD3^+^ T-cells, higher secretion of IFN-γ, upregulated levels of Caspase-3, heat-shock protein 70, HLA-DR, and PD-L1 within the tumor microenvironment (TME). Out of the six patients, two showed prolonged therapeutic responses (>23 months) without further therapies; however, four had to undergo additional surgical procedures for removal of accumulated SPIONs (Grauer et al., 2019).

Gadolinium-chelated polysiloxane based nanoparticles (AGuIX) have shown promising results as a radiosensitizer both *in vitro* (Mowat et al., 2011) and *in vivo* (Sancey et al., 2015; Kotb et al., 2016). It has demonstrated great potential as a theranostic agent for enhancing MRI contrast and as a cytotoxic agent when conjugated with radiotherapy (Sancey et al., 2014). These findings have motivated several clinical studies for the safe application of these NPs in human cancer patients (Lux et al., 2019). An ongoing NANO-RAD trial (NCT02820454) utilizing gadolinium-chelated polysiloxane based nanoparticles (AGuIX) as a theranostic as well as a radiosensitizer agent has recently completed the recruitment process. This phase I study will consist of about 15 participants and is aimed at investigating the biosafety, dose tolerance, and toxicology of the AGuIX NPs when applied in combination with standard whole-brain radiotherapy (WBRT). The administration of Nps will be done intravenously (IV) followed by a magnetic resonance imaging (MRI) scan to analyze the distribution of NPs. The patients will be subjected to WBRT starting 4 h after IV administration, 5 days a week till 2 weeks of treatment. The pharmacological studies of AGuIX Nps will be performed at regular intervals by drawing blood from the patients. Phase II of this trial (NANORAD2; NCT03818386) is also currently under the recruitment stage. Another Phase II clinical trial to evaluate the clinical impact of AGuIX in combination with fractionated stereotactic radiotherapy (NANOSTEREO; NCT04094077) is currently recruiting. Additionally, another Phase I clinical trial (NANOCOL; NCT03308604) for patients with locally advanced cervical cancer is currently in the recruitment phase. The trial is based on evaluating the biosafety and dosage tolerance of AGuIX NPs in conjugation with radiation and cisplatin.

Another interesting research study (WDVAX; NCT01753089) which is based on the formulation of a personalized melanoma vaccine is currently under Phase I of clinical trials. The vaccine will contain tumor lysate obtained from the patient’s own melanoma tumor cells combined with other immunostimulatory agents such as CpG and granulocyte-macrophage colony-stimulating factor (GM-CSF). These components will be combined together in a polymeric biomaterial scaffold consisting of PLGA. The trial consists of 23 participants with metastatic melanoma and is aimed at establishing the biosafety and efficacy of the scaffold-based vaccine in modulating the patient’s own immune system to build effective antitumor responses against the melanoma tumor cells (Dolgin, 2013; Singh and Peppas, 2014).

Since, proteins and the immune cells are the first to encounter NMs upon administration into an *in vivo* biological system, therefore, prior to any clinical application, it is essential to critically assess the nature of interactions of NMs with the cells and the proteins of the living system so as to ensure that the benefits outweigh the limitations of any NM-based clinical therapy. However, majority of the present clinical trials tend to emphasize only on the therapeutic aspect of the NM-based treatments while leaving out the biosafety concerns involved. Also, there is a dire need to develop a unified and standardized procedure for determining the biosafety of NMs during research and the clinical studies in order to minimize adverse effects during human trials and ensuring that more and more NM-based therapies successfully attain clinical translation.

## Conclusion

Immunomodulation based cancer therapies rely on altering the body’s own defenses to fight back and eradicate its enemy, cancer. The targeted therapeutic effect in minimal concentrations while causing minimum/no side-effects is the major reason for the success of this approach. However, due to the heterogeneous cellular composition of tumors, individual cancer therapies, especially the ones based on conventional approaches like surgery, radiation, and chemotherapy have shown limited success. When used in isolation, these treatments do not provide a curative measure for cancer. Therefore, to enhance the efficacy of the treatment and achieve better survivability, it is necessary to explore combinatorial cancer therapies. The unintended and unavoidable interactions of nanomaterials with the biological systems including the immune system is a major hurdle for tapping the optimum therapeutic effect. Due to this, only a sparse number of immunotherapies have attained successful clinical application. Thus, the future advancements in applying immunomodulatory nanomaterials for cancer therapeutics should be majorly focused on taking into consideration the biosafety aspects of the use of nanomaterial. Additionally, favorable aspect for the future cancer therapies will be based on delivery of more than one active agent so as to accommodate simultaneous diagnosis, targeting, treatment and monitoring. Such immunomodulatory-based cancer therapeutics will exhibit many promising results and improve survivability when used in conjugation with other techniques to create an effective anti-cancer regimen. Another aspect for potential research in this field can be towards achieving precisely targeted delivery and stimuli-responsive release of immunomodulatory therapeutic agents.

## Glossary

Nanomaterials (NMs): NMs are a class of materials that possess at least one dimension within the range of 1–100 nm
Immunity: The inherent ability of an organism to avert pathogens and diseases by recognition and subsequent neutralization or elimination of foreign entities by the organism’s immune system
Immunomodulation: The process of altering the host’s immune system and its responses
Immuno-stimulation: The process of enhancing the ability of the body to evoke a strong and robust immune response against an antigen
Immuno-suppression: The diminished ability of the body to induce an immune response due to a lack of immune cells such as T and/or B lymphocyte

## References

[B1] Abbott M. Ustoyev Y. (2019). Cancer and the immune system: the history and background of immunotherapy. Semin. Oncol. Nurs. 10, 150923. 10.1016/j.soncn.2019.08.002 31526550

[B2] Al-Tameemi M. Chaplain M. d'Onofrio A. (2012). Evasion of tumours from the control of the immune system: consequences of brief encounters. Biol. Direct 7, 31. 10.1186/1745-6150-7-31 23009638 PMC3582466

[B3] Alsaleh N. B. Brown J. M. (2018). Immune responses to engineered nanomaterials: current understanding and challenges. Curr. Opin. Toxicol. 10, 8–14. 10.1016/j.cotox.2017.11.011 29577105 PMC5863933

[B4] Alyassin Y. Sayed E. G. Mehta P. Ruparelia K. Arshad M. S. Rasekh M. (2020). Application of mesoporous silica nanoparticles as drug delivery carriers for chemotherapeutic agents. Drug Discov. Today 25, 1513–1520. 10.1016/j.drudis.2020.06.006 32561300

[B5] Aquib M. Farooq M. A. Banerjee P. Akhtar F. Filli M. S. Boakye-Yiadom K. O. (2019). Targeted and stimuli-responsive mesoporous silica nanoparticles for drug delivery and theranostic use. J. Biomed. Mater. Res. 107 (107), 2643–2666. 10.1002/jbm.a.36770 31390141

[B6] Asadi N. Davaran S. Panahi Y. Hasanzadeh A. Malakootikhah J. Fallah Moafi H. (2017). Application of nanostructured drug delivery systems in immunotherapy of cancer: a review. Artif. Cells Nanomed. Biotechnol. 45, 18–23. 10.1080/21691401.2016.1178136 27196810

[B7] Babiker H. M. McBride A. Newton M. Boehmer L. M. Drucker A. G. Gowan M. (2018). Cardiotoxic effects of chemotherapy: a review of both cytotoxic and molecular targeted oncology therapies and their effect on the cardiovascular system. Crit. Rev. Oncol. Hematol. 126, 186–200. 10.1016/j.critrevonc.2018.03.014 29759560

[B8] Baetke S. C. Lammers T. Kiessling F. (2015). Applications of nanoparticles for diagnosis and therapy of cancer. Br. J. Radiol. 88, 20150207. 10.1259/bjr.20150207 25969868 PMC4630860

[B9] Balakrishnan S. Mukherjee S. Das S. Bhat F. A. Raja Singh P. Patra C. R. (2017). Gold nanoparticles-conjugated quercetin induces apoptosis via inhibition of EGFR/PI3K/Akt-mediated pathway in breast cancer cell lines (MCF-7 and MDA-MB-231). Cell Biochem. Funct. 35, 217–231. 10.1002/cbf.3266 28498520

[B10] Ballas Z. K. (2018). The 2018 Nobel Prize in Physiology or Medicine: an exemplar of bench to bedside in immunology. J. Allergy Clin. Immunol. 12 (142), 1752–1753. 10.1016/j.jaci.2018.10.021 30539724

[B11] Bartneck M. (2017). Immunomodulatory nanomedicine. Macromol. Biosci. 17, 17. 10.1002/mabi.201700021 28383783

[B12] Belardelli F. Ferrantini M. (2002). Cytokines as a link between innate and adaptive antitumor immunity. Trends Immunol. 23, 201–208. 10.1016/s1471-4906(02)02195-6 11923115

[B13] Bleeker E. A. de Jong W. H. Geertsma R. E. Groenewold M. Heugens E. H. Koers-Jacquemijns M. (2013). Considerations on the EU definition of a nanomaterial: science to support policy making. Regul. Toxicol. Pharmacol. 65, 119–125. 10.1016/j.yrtph.2012.11.007 23200793

[B14] Bonaventura P. Shekarian T. Alcazer V. Valladeau-Guilemond J. Valsesia-Wittmann S. Amigorena S. (2019). Cold tumors: a therapeutic challenge for immunotherapy. Front. Immunol. 10, 168. 10.3389/fimmu.2019.00168 30800125 PMC6376112

[B15] Bonilla F. A. Oettgen H. C. (2010). Adaptive immunity. J. Allergy Clin. Immunol. 125, S33–S40. 10.1016/j.jaci.2009.09.017 20061006

[B16] Braff M. H. Gallo R. L. (2006). Antimicrobial peptides: an essential component of the skin defensive barrier. Curr. Top. Microbiol. Immunol. 306, 91–110. 10.1007/3-540-29916-5_4 16909919

[B17] Broide D. H. (2009). Immunomodulation of allergic disease. Annu. Rev. Med. 60, 279–291. 10.1146/annurev.med.60.041807.123524 19630573 PMC2779001

[B18] Buabeid M. A. Arafa E. A. Murtaza G. (2020). Emerging prospects for nanoparticle-enabled cancer immunotherapy. J. Immunol. Res. 2020, 9624532. 10.1155/2020/9624532 32377541 PMC7199570

[B19] Calì B. Molon B. Viola A. (2017). Tuning cancer fate: the unremitting role of host immunity. Open Biol. 7, 7. 10.1098/rsob.170006 PMC5413907 28404796

[B20] Carballido J. M. Regairaz C. Rauld C. Raad L. Picard D. Kammüller M. (2020). The emerging jamboree of transformative therapies for autoimmune diseases. Front. Immunol. 11, 472. 10.3389/fimmu.2020.00472 32296421 PMC7137386

[B21] Chan C. W. Crafton E. Fan H. N. Flook J. Yoshimura K. Skarica M. (2006). Interferon-producing killer dendritic cells provide a link between innate and adaptive immunity. Nat. Med. 12, 207–213. 10.1038/nm1352 16444266

[B22] Chang R. Wang P. Y. Tseng C. L. (2018). New combination/application of polymer-based nanoparticles for biomedical engineering. Adv. Exp. Med. Biol. 1078, 271–290. 10.1007/978-981-13-0950-2_14 30357628

[B23] Chen D. S. Mellman I. (2017). Elements of cancer immunity and the cancer-immune set point. Nature. 541, 321–330. 10.1038/nature21349 28102259

[B24] Chen D. S. Mellman I. (2013). Oncology meets immunology: the cancer-immunity cycle. Immunity. 39, 1–10. 10.1016/j.immuni.2013.07.012 23890059

[B25] Chen H. Zhang W. Zhu G. Xie J. Chen X. (2017). Rethinking cancer nanotheranostics. Nat Rev Mater. 2, 24. 10.1038/natrevmats.2017.24 PMC5654564 29075517

[B26] Chen Y. S. Hung Y. C. Liau I. Huang G. S. (2009). Assessment of the in vivo toxicity of gold nanoparticles. Nanoscale Res. Lett. 4, 858–864. 10.1007/s11671-009-9334-6 20596373 PMC2894102

[B27] Cheng Y. Dai Q. Morshed R. A. Fan X. Wegscheid M. L. Wainwright D. A. (2014). Blood-brain barrier permeable gold nanoparticles: an efficient delivery platform for enhanced malignant glioma therapy and imaging. Small. 10, 5137–5150. 10.1002/smll.201400654 25104165 PMC4268041

[B28] Chidambaram M. Manavalan R. Kathiresan K. (2011). Nanotherapeutics to overcome conventional cancer chemotherapy limitations. J. Pharm. Pharm. Sci. 14, 67–77. 10.18433/j30c7d 21501554

[B29] Chircop M. Speidel D. (2014). Cellular stress responses in cancer and cancer therapy. Front. Oncol. 4, 304. 10.3389/fonc.2014.00304 25401089 PMC4212619

[B30] Cisterna B. A. Kamaly N. Choi W. I. Tavakkoli A. Farokhzad O. C. Vilos C. (2016). Targeted nanoparticles for colorectal cancer. Nanomedicine. 11, 2443–2456. 10.2217/nnm-2016-0194 27529192 PMC5619175

[B31] Coates M. Blanchard S. MacLeod A. S. (2018). Innate antimicrobial immunity in the skin: a protective barrier against bacteria, viruses, and fungi. PLoS Pathog. 14 (14), e1007353. 10.1371/journal.ppat.1007353 30522130 PMC6283644

[B32] Coley W. B. (1991). The treatment of malignant tumors by repeated inoculations of erysipelas. With a report of ten original cases. Clin. Orthop. Relat. Res. 1893, 3–11. 1984929

[B33] Comparetti E. J. Pedrosa V. A. Kaneno R. (2018). Carbon nanotube as a tool for fighting cancer. Bioconjug. Chem. 3, 709–718. 10.1021/acs.bioconjchem.7b00563 29072905

[B34] Cooper E. L. (2016). Commentary: blurring borders: innate immunity with adaptive features. Front. Microbiol. 7, 358. 10.3389/fmicb.2016.00358 27047472 PMC4805646

[B35] Cosentino D. Piro F. (2018). Hyaluronic acid for treatment of the radiation therapy side effects: a systematic review. Eur. Rev. Med. Pharmacol. Sci. 11 (22), 7562–7572. 10.26355/eurrev_201811_16298 30468506

[B36] Curiel T. J. (2007). Tregs and rethinking cancer immunotherapy. J. Clin. Invest. 117, 1167–1174. 10.1172/JCI31202 17476346 PMC1857250

[B37] Curti B. D. Kovacsovics-Bankowski M. Morris N. Walker E. Chisholm L. Floyd K. (2013). OX40 is a potent immune-stimulating target in late-stage cancer patients. Cancer Res. 73, 7189–7198. 10.1158/0008-5472.CAN-12-4174 24177180 PMC3922072

[B38] D'Errico G. Machado H. L. Sainz B. (2017). A current perspective on cancer immune therapy: step-by-step approach to constructing the magic bullet. Clin. Transl. Med. 6, 3. 10.1186/s40169-016-0130-5 28050779 PMC5209322

[B39] Dai Z. Leung H. M. Lo P. K. (2017). Stimuli-responsive self-assembled DNA nanomaterials for biomedical applications. Small. 13, 13. 10.1002/smll.201602881 28005298

[B40] de Visser K. E. Eichten A. Coussens L. M. (2006). Paradoxical roles of the immune system during cancer development. Nat. Rev. Cancer. 6, 24–37. 10.1038/nrc1782 16397525

[B41] Decker W. K. Safdar A. (2009). Bioimmunoadjuvants for the treatment of neoplastic and infectious disease: Coley's legacy revisited. Cytokine Growth Factor Rev. 20, 271–281. 10.1016/j.cytogfr.2009.07.004 19656718

[B42] Demir E. (2021). A review on nanotoxicity and nanogenotoxicity of different shapes of nanomaterials. J. Appl. Toxicol. 41, 118–147. 10.1002/jat.4061 33111384

[B43] Demir E. (2020). An *in vivo* study of nanorod, nanosphere, and nanowire forms of titanium dioxide using *Drosophila melanogaster*: toxicity, cellular uptake, oxidative stress, and DNA damage. J. Toxicol. Environ. Health 83, 456–469. 10.1080/15287394.2020.1777236 32515692

[B44] Desai N. Trieu V. Yao Z. Louie L. Ci S. Yang A. (2006). Increased antitumor activity, intratumor paclitaxel concentrations, and endothelial cell transport of cremophor-free, albumin-bound paclitaxel, ABI-007, compared with cremophor-based paclitaxel. Clin. Cancer Res. 12, 1317–1324. 10.1158/1078-0432.CCR-05-1634 16489089

[B45] Dhivya S. Ajita J. Selvamurugan N. (2015). Metallic nanomaterials for bone tissue engineering. J. Biomed. Nanotechnol. 11, 1675–1700. 10.1166/jbn.2015.2115 26502634

[B46] Dhodapkar K. M. (2019). Autoimmune complications of cancer immunotherapy. Curr. Opin. Immunol. 12 (61), 54–59. 10.1016/j.coi.2019.08.004 PMC7968474 31557690

[B47] Dickneite G. Kaspereit F. Sedlacek H. H. (1984). Stimulation of cell-mediated immunity by bestatin correlates with reduction of bacterial persistence in experimental chronic *Salmonella typhimurium* infection. Infect. Immun. 44, 168–174. 10.1128/IAI.44.1.168-174.1984 6368392 PMC263488

[B48] Diou O. Tsapis N. Fattal E. (2012). Targeted nanotheranostics for personalized cancer therapy. Expert Opin. Drug Deliv. 9, 1475–1487. 10.1517/17425247.2012.736486 23092183

[B49] Dittmer U. Olbrich A. R. (2003). Treatment of infectious diseases with immunostimulatory oligodeoxynucleotides containing CpG motifs. Curr. Opin. Microbiol. 6, 472–477. 10.1016/j.mib.2003.09.007 14572539

[B50] Dolgin E. (2013). Cancer vaccines: material breach. Nature 504, S16–S17. 10.1038/504S16a Dec 24352360

[B51] Draghi A. Chamberlain C. A. Furness A. Donia M. (2019). Acquired resistance to cancer immunotherapy. Semin. Immunopathol. 41, 31–40. 10.1007/s00281-018-0692-y 29968044

[B52] Drews J. (1980). A role for immune stimulation in the treatment of microbial infections?. Infection 8, 2–4. 10.1007/BF01677390 6966257

[B53] Duan Q. Zhang H. Zheng J. Zhang L. (2020). Turning cold into hot: firing up the tumor microenvironment. Trends Cancer 7 (6), 605–618. 10.1016/j.trecan.2020.02.022 32610070

[B54] Dunn G. P. Bruce A. T. Ikeda H. Old L. J. Schreiber R. D. (2002). Cancer immunoediting: from immunosurveillance to tumor escape. Nat. Immunol. 3, 991–998. 10.1038/ni1102-991 12407406

[B55] Dunn G. P. Koebel C. M. Schreiber R. D. (2006). Interferons, immunity and cancer immunoediting. Nat. Rev. Immunol. 6, 836–848. 10.1038/nri1961 17063185

[B56] Dunn G. P. Old L. J. Schreiber R. D. (2004). The three Es of cancer immunoediting. Annu. Rev. Immunol. 22, 329–360. 10.1146/annurev.immunol.22.012703.104803 15032581

[B57] Elzey B. D. Sprague D. L. Ratliff T. L. (2005). The emerging role of platelets in adaptive immunity. Cell. Immunol. 238, 1–9. 10.1016/j.cellimm.2005.12.005 16442516

[B58] Elzey B. D. Tian J. Jensen R. J. Swanson A. K. Lees J. R. Lentz S. R. (2003). Platelet-mediated modulation of adaptive immunity. A communication link between innate and adaptive immune compartments. Immunity 19, 9–19. 10.1016/s1074-7613(03)00177-8 12871635

[B59] Ephrem A. Misra N. Hassan G. Dasgupta S. Delignat S. Duong Van Huyen J. P. (2005). Immunomodulation of autoimmune and inflammatory diseases with intravenous immunoglobulin. Clin. Exp. Med. 5, 135–140. 10.1007/s10238-005-0079-y 16362793

[B60] Fam S. Y. Chee C. F. Yong C. Y. Ho K. L. Mariatulqabtiah A. R. Tan W. S. (2020). Stealth coating of nanoparticles in drug-delivery systems. Nanomaterials 10, 787. 10.3390/nano10040787 PMC7221919 32325941

[B61] Fehniger T. A. Cooper M. A. Nuovo G. J. Cella M. Facchetti F. Colonna M. (2003). CD56bright natural killer cells are present in human lymph nodes and are activated by T cell-derived IL-2: a potential new link between adaptive and innate immunity. Blood 101, 3052–3057. 10.1182/blood-2002-09-2876 12480696

[B62] Feng X. Xu W. Li Z. Song W. Ding J. Chen X. (2019). Immunomodulatory nanosystems. Adv. Sci. 6, 1900101. 10.1002/advs.201900101 PMC6724480 31508270

[B63] Ferrari S. Rovati B. Porta C. Alessandrino P. E. Bertolini A. Collovà E. (2003). Lack of dendritic cell mobilization into the peripheral blood of cancer patients following standard- or high-dose chemotherapy plus granulocyte-colony stimulating factor. Cancer Immunol. Immunother. 52, 359–366. 10.1007/s00262-002-0365-4 12664135 PMC11033038

[B64] Finlay B. B. McFadden G. (2006). Anti-immunology: evasion of the host immune system by bacterial and viral pathogens. Cell 124, 767–782. 10.1016/j.cell.2006.01.034 16497587

[B65] Fornaguera C. Feiner-Gracia N. Calderó G. García-Celma M. J. Solans C. (2015). Galantamine-loaded PLGA nanoparticles, from nano-emulsion templating, as novel advanced drug delivery systems to treat neurodegenerative diseases. Nanoscale 7, 12076–12084. 10.1039/c5nr03474d 26118655

[B66] Francis A. P. Devasena T. (2018). Toxicity of carbon nanotubes: a review. Toxicol. Ind. Health 34, 200–210. 10.1177/0748233717747472 29506458

[B67] Fukuda A. Tahara K. Hane Y. Matsui T. Sasaoka S. Hatahira H. (2017). Comparison of the adverse event profiles of conventional and liposomal formulations of doxorubicin using the FDA adverse event reporting system. PLoS One 12, e0185654. 10.1371/journal.pone.0185654 28953936 PMC5617225

[B68] Galstyan A. Markman J. L. Shatalova E. S. Chiechi A. Korman A. J. Patil R. (2019). Blood-brain barrier permeable nano immunoconjugates induce local immune responses for glioma therapy. Nat. Commun. 08, 3850. 10.1038/s41467-019-11719-3 PMC6713723 31462642

[B69] Gao W. Xiong Y. Li Q. Yang H. (2017). Inhibition of toll-like receptor signaling as a promising therapy for inflammatory diseases: a journey from molecular to nano therapeutics. Front. Physiol. 8, 508. 10.3389/fphys.2017.00508 28769820 PMC5516312

[B70] García-Martínez E. Smith M. Buqué A. Aranda F. de la Peña F. A. Ivars A. (2018). Trial Watch: Immunostimulation with recombinant cytokines for cancer therapy. Oncoimmunology 7, e1433982. 29872569 10.1080/2162402X.2018.1433982 PMC5980390

[B71] Gheibi Hayat S. M. Bianconi V. Pirro M. Sahebkar A. (2019). Stealth functionalization of biomaterials and nanoparticles by CD47 mimicry. Int. J. Pharm. 569, 118628. 10.1016/j.ijpharm.2019.118628 31421198

[B72] Gilabert-Oriol R. Ryan G. M. Leung A. W. Y. Firmino N. S. Bennewith K. L. Bally M. B. (2018). Liposomal formulations to modulate the tumour microenvironment and antitumour immune response. Int. J. Mol. Sci. 19 (10), 2922. 10.3390/ijms19102922 PMC6213379 30261606

[B73] Grabowski M. M. Sankey E. W. Ryan K. J. Chongsathidkiet P. Lorrey S. J. Wilkinson D. S. (2020). Immune suppression in gliomas. J. Neuro Oncol. 151 (1), 3–12. 10.1007/s11060-020-03483-y PMC7843555 32542437

[B74] Grauer O. Jaber M. Hess K. Weckesser M. Schwindt W. Maring S. (2019). Combined intracavitary thermotherapy with iron oxide nanoparticles and radiotherapy as local treatment modality in recurrent glioblastoma patients. J. Neuro Oncol. 141, 83–94. 10.1007/s11060-018-03005-x PMC6341053 30506500

[B75] Gregersen P. K. Behrens T. W. (2006). Genetics of autoimmune diseases--disorders of immune homeostasis. Nat. Rev. Genet. 7, 917–928. 10.1038/nrg1944 17139323

[B76] Hall M. W. Joshi I. Leal L. Ooi E. E. (2020). Immune modulation in COVID-19: strategic considerations for personalized therapeutic intervention. Clin. Infect. Dis. ciaa904. 10.1093/cid/ciaa904 32604407 PMC7337699

[B77] Hapuarachchige S. Artemov D. (2020). Theranostic pretargeting drug delivery and imaging platforms in cancer precision medicine. Front. Oncol. 10, 1131. 10.3389/fonc.2020.01131 32793481 PMC7387661

[B78] Hegde P. S. Chen D. S. (2020). Top 10 challenges in cancer immunotherapy. Immunity 52, 17–35. 10.1016/j.immuni.2019.12.011 31940268

[B79] Houghton A. N. (1994). Cancer antigens: immune recognition of self and altered self. J. Exp. Med. 180, 1–4. 10.1084/jem.180.1.1 8006576 PMC2191545

[B80] Iglesias N. Galbis E. Díaz-Blanco M. J. de-Paz M. V. Galbis J. A. (2018). Loading studies of the anticancer drug camptothecin into dual stimuli-sensitive nanoparticles. Stability scrutiny. Int. J. Pharm. 550, 429–438. 10.1016/j.ijpharm.2018.08.026 30196142

[B81] Immunotherapies for Autoimmune Diseases (2019). Cancer immunotherapies repurposed for use in autoimmunity. Nat. Biomed. Eng. 3 (4), 247. 10.1038/s41551-019-0394-3 30952989

[B82] Ingraham N. E. Lotfi-Emran S. Thielen B. K. Techar K. Morris R. S. Holtan S. G. (2020). Immunomodulation in COVID-19. Lancet Respir. Med. 6, 544–546. 10.1016/S2213-2600(20)30226-5 PMC7198187 32380023

[B83] Irvine D. J. Swartz M. A. Szeto G. L. (2013). Engineering synthetic vaccines using cues from natural immunity. Nat. Mater. 12, 978–990. 10.1038/nmat3775 24150416 PMC3928825

[B84] Iwasaki A. Medzhitov R. (2015). Control of adaptive immunity by the innate immune system. Nat. Immunol. 16, 343–353. 10.1038/ni.3123 25789684 PMC4507498

[B85] Jena L. McErlean E. McCarthy H. (2020). Delivery across the blood-brain barrier: nanomedicine for glioblastoma multiforme. Drug Deliv. Transl. Res. 10, 304–318. 10.1007/s13346-019-00679-2 31728942 PMC7066289

[B86] Jiang B. P. Zhou B. Lin Z. Liang H. Shen X. C. (2019). Recent advances in carbon nanomaterials for cancer phototherapy. Chemistry 25, 3993–4004. 10.1002/chem.201804383 30328167

[B87] Jiang Q. Luo Z. Men Y. Yang P. Peng H. Guo R. (2017). Red blood cell membrane-camouflaged melanin nanoparticles for enhanced photothermal therapy. Biomaterials 143, 29–45. 10.1016/j.biomaterials.2017.07.027 28756194

[B88] Jo S. D. Ku S. H. Won Y. Y. Kim S. H. Kwon I. C. (2016). Targeted nanotheranostics for future personalized medicine: recent progress in cancer therapy. Theranostics 6, 1362–1377. 10.7150/thno.15335 27375785 PMC4924505

[B89] Kashi T. S. Eskandarion S. Esfandyari-Manesh M. Marashi S. M. Samadi N. Fatemi S. M. (2012). Improved drug loading and antibacterial activity of minocycline-loaded PLGA nanoparticles prepared by solid/oil/water ion pairing method. Int. J. Nanomed. 7, 221–234. 10.2147/IJN.S27709 PMC3263414 22275837

[B90] Kazemi T. Younesi V. Jadidi-Niaragh F. Yousefi M. (2016). Immunotherapeutic approaches for cancer therapy: an updated review. Artif Cells Nanomed. Biotechnol. 44, 769–779. 10.3109/21691401.2015.1019669 25801036

[B91] Kelly J. Leonard W. J. (2003). Immune deficiencies due to defects in cytokine signaling. Curr. Allergy Asthma Rep. 3, 396–401. 10.1007/s11882-003-0073-y 12906775

[B92] Kitamura T. Qian B. Z. Pollard J. W. (2015). Immune cell promotion of metastasis. Nat. Rev. Immunol. 15, 73–86. 10.1038/nri3789 25614318 PMC4470277

[B93] Knochelmann H. M. Dwyer C. J. Bailey S. R. Amaya S. M. Elston D. M. Mazza-McCrann J. M. (2018). When worlds collide: Th17 and Treg cells in cancer and autoimmunity. Cell. Mol. Immunol. 15, 458–469. 10.1038/s41423-018-0004-4 29563615 PMC6068176

[B94] Kotb S. Detappe A. Lux F. Appaix F. Barbier E. L. Tran V. L. (2016). Gadolinium-based nanoparticles and radiation therapy for multiple brain melanoma metastases: proof of concept before phase I trial. Theranostics 6, 418–427. 10.7150/thno.14018 26909115 PMC4737727

[B95] Kroemer G. Zitvogel L. (2018). Cancer immunotherapy in 2017: the breakthrough of the microbiota. Nat. Rev. Immunol. 18, 87–88. 10.1038/nri.2018.4 29379189

[B96] Kubackova J. Zbytovska J. Holas O. (2020). Nanomaterials for direct and indirect immunomodulation: a review of applications. Eur. J. Pharm. Sci. 142, 105139. 10.1016/j.ejps.2019.105139 31704342

[B97] Kwiatkowski A. J. Stewart J. M. Cho J. J. Avram D. Keselowsky B. G. (2020). Nano and microparticle emerging strategies for treatment of autoimmune diseases: multiple sclerosis and type 1 diabetes. Adv. Healthc. Mater. 6 (9), e2000164. 10.1002/adhm.202000164 PMC7588284 32519501

[B98] La-Beck N. M. Liu X. Wood L. M. (2019). Harnessing liposome interactions with the immune system for the next breakthrough in cancer drug delivery. Front. Pharmacol. 10, 220. 10.3389/fphar.2019.00220 30914953 PMC6422978

[B99] Lakshmi Narendra B. Eshvendar Reddy K. Shantikumar S. Ramakrishna S. (2013). Immune system: a double-edged sword in cancer. Inflamm. Res. 62, 823–834. 10.1007/s00011-013-0645-9 23868500

[B100] Lee C. Hwang H. S. Lee S. Kim B. Kim J. O. Oh K. T. (2017). Rabies virus-inspired silica-coated gold nanorods as a photothermal therapeutic platform for treating brain tumors. Adv. Mater. Weinheim 29, 5563. 10.1002/adma.201605563 28134459

[B101] Lee S. Khang D. Kim S. H. (2015). High dispersity of carbon nanotubes diminishes immunotoxicity in spleen. Int. J. Nanomed. 10, 2697–2710. 10.2147/IJN.S80836 PMC4388092 25878502

[B102] Lei X. Lei Y. Li J. K. Du W. X. Li R. G. Yang J. (2020). Immune cells within the tumor microenvironment: biological functions and roles in cancer immunotherapy. Cancer Lett. 2, 126–133. 10.1016/j.canlet.2019.11.009 31730903

[B103] Li B. Wang F. Gui L. He Q. Yao Y. Chen H. (2018). The potential of biomimetic nanoparticles for tumor-targeted drug delivery. Nanomedicine 13, 2099–2118. 10.2217/nnm-2018-0017 30226404

[B104] Li C. Han X. (2020). Melanoma cancer immunotherapy using PD-L1 siRNA and imatinib promotes cancer-immunity cycle. Pharm. Res. 37, 109. 10.1007/s11095-020-02838-4 32476052

[B105] Li C. W. Li L. L. Chen S. Zhang J. X. Lu W. L. (2020). Antioxidant nanotherapies for the treatment of inflammatory diseases. Front. Bioeng. Biotechnol. 8, 200. 10.3389/fbioe.2020.00200 32258013 PMC7093330

[B106] Li J. Van Valkenburgh J. Hong X. Conti P. S. Zhang X. Chen K. (2019). Small molecules as theranostic agents in cancer immunology. Theranostics 9, 7849–7871. 10.7150/thno.37218 31695804 PMC6831453

[B107] Li L. Neaves W. B. (2006). Normal stem cells and cancer stem cells: the niche matters. Cancer Res. 66, 4553–4557. 10.1158/0008-5472.CAN-05-3986 16651403

[B108] Li L. Wang J. Kong H. Zeng Y. Liu G. (2018). Functional biomimetic nanoparticles for drug delivery and theranostic applications in cancer treatment. Sci. Technol. Adv. Mater. 19, 771–790. 10.1080/14686996.2018.1528850 30815042 PMC6383616

[B109] Li Q. Zhang D. Zhang J. Jiang Y. Song A. Li Z. (2019). A three-in-one immunotherapy nanoweapon via Cascade-amplifying cancer-immunity cycle against tumor metastasis, relapse, and postsurgical regrowth. Nano Lett. 19, 6647–6657. 10.1021/acs.nanolett.9b02923 31409072

[B110] Li S. Amat D. Peng Z. Vanni S. Raskin S. De Angulo G. (2016). Transferrin conjugated nontoxic carbon dots for doxorubicin delivery to target pediatric brain tumor cells. Nanoscale 8, 16662–16669. 10.1039/c6nr05055g 27714111

[B111] Li T. Qin X. Li Y. Shen X. Li S. Yang H. (2020). Cell membrane coated-biomimetic nanoplatforms toward cancer theranostics. Front. Bioeng. Biotechnol. 8, 371. 10.3389/fbioe.2020.00371 32411690 PMC7202082

[B112] Li T. Shi S. Goel S. Shen X. Xie X. Chen Z. (2019). Recent advancements in mesoporous silica nanoparticles towards therapeutic applications for cancer. Acta Biomater. 4, 1–13. 10.1016/j.actbio.2019.02.031 30797106

[B113] Liu Y. Cao X. (2016). Immunosuppressive cells in tumor immune escape and metastasis. J Mol Med. 94, 509–522. 10.1007/s00109-015-1376-x 26689709

[B114] Liu Y. Wang X. Hussain M. Lv M. Dong X. Wang T. (2018). Theranostics applications of nanoparticles in cancer immunotherapy. Med. Sci. 6. 10.3390/medsci6040100 PMC6313674 30424010

[B115] Liu Z. Jiang W. Nam J. Moon J. J. Kim B. Y. S. (2018). Immunomodulating nanomedicine for cancer therapy. Nano Lett. 18 (18), 6655–6659. 10.1021/acs.nanolett.8b02340 30185039 PMC6238186

[B116] Liyanage P. Y. Zhou Y. Al-Youbi A. O. Bashammakh A. S. El-Shahawi M. S. Vanni S. (2020). Pediatric glioblastoma target-specific efficient delivery of gemcitabine across the blood-brain barrier via carbon nitride dots. Nanoscale. 12, 7927–7938. 10.1039/d0nr01647k 32232249

[B117] Locy H. de Mey S. de Mey W. De Ridder M. Thielemans K. Maenhout S. K. (2018). Immunomodulation of the tumor microenvironment: turn foe into friend. Front. Immunol. 9, 2909. 10.3389/fimmu.2018.02909 30619273 PMC6297829

[B118] Lorente C. Arias J. L. Cabeza L. Ortiz R. Prados J. C. Melguizo C. (2018). Nano-engineering of biomedical prednisolone liposomes: evaluation of the cytotoxic effect on human colon carcinoma cell lines. J. Pharm. Pharmacol. 70, 488–497. 10.1111/jphp.12882 29380384

[B119] Lux F. Tran V. L. Thomas E. Dufort S. Rossetti F. Martini M. (2019). AGuIX® from bench to bedside-Transfer of an ultrasmall theranostic gadolinium-based nanoparticle to clinical medicine. Br. J. Radiol. 92, 20180365. 10.1259/bjr.20180365 30226413 PMC6435081

[B120] Makovec T. (2019). Cisplatin and beyond: molecular mechanisms of action and drug resistance development in cancer chemotherapy. Radiol. Oncol. 03, 148–158. 10.2478/raon-2019-0018 PMC6572495 30956230

[B121] Martinon F. Tschopp J. (2004). Inflammatory caspases: linking an intracellular innate immune system to autoinflammatory diseases. Cell. 117, 561–574. 10.1016/j.cell.2004.05.004 15163405

[B122] Melero I. Rouzaut A. Motz G. T. Coukos G. (2014). T-cell and NK-cell infiltration into solid tumors: a key limiting factor for efficacious cancer immunotherapy. Cancer Discov. 4, 522–526. 10.1158/2159-8290.CD-13-0985 24795012 PMC4142435

[B123] Meng H. Chen Z. Xing G. Yuan H. Chen C. Zhao F. (2007). Ultrahigh reactivity provokes nanotoxicity: explanation of oral toxicity of nano-copper particles. Toxicol. Lett. 175, 102–110. 10.1016/j.toxlet.2007.09.015 18024012

[B124] Met Ö. Jensen K. M. Chamberlain C. A. Donia M. Svane I. M. (2019). Principles of adoptive T cell therapy in cancer. Semin. Immunopathol. 41, 49–58. 10.1007/s00281-018-0703-z 30187086

[B125] Miliotou A. N. Papadopoulou L. C. (2018). CAR T-cell therapy: a new era in cancer immunotherapy. Curr. Pharm. Biotechnol. 19, 5–18. 10.2174/1389201019666180418095526 29667553

[B126] Mittal D. Gubin M. M. Schreiber R. D. Smyth M. J. (2014). New insights into cancer immunoediting and its three component phases--elimination, equilibrium and escape. Curr. Opin. Immunol. 27, 16–25. 10.1016/j.coi.2014.01.004 24531241 PMC4388310

[B127] Mohme M. Riethdorf S. Pantel K. (2017). Circulating and disseminated tumour cells - mechanisms of immune surveillance and escape. Nat. Rev. Clin. Oncol. 14, 155–167. 10.1038/nrclinonc.2016.144 27644321

[B128] Moser M. Leo O. (2010). Key concepts in immunology. Vaccine. 28 (Suppl. 3), C2–C13. 10.1016/j.vaccine.2010.07.022 20713253

[B129] Mowat P. Mignot A. Rima W. Lux F. Tillement O. Roulin C. (2011). *In vitro* radiosensitizing effects of ultrasmall gadolinium based particles on tumour cells. J. Nanosci. Nanotechnol. 11, 7833–7839. 10.1166/jnn.2011.4725 22097494

[B130] Muenst S. Läubli H. Soysal S. D. Zippelius A. Tzankov A. Hoeller S. (2016). The immune system and cancer evasion strategies: therapeutic concepts. J. Intern. Med. 279, 541–562. 10.1111/joim.12470 26748421

[B131] Mühlberger M. Janko C. Unterweger H. Friedrich R. P. Friedrich B. Band J. (2019). Functionalization of T lymphocytes with citrate-coated superparamagnetic iron oxide nanoparticles for magnetically controlled immune therapy. Int. J. Nanomed. 14, 8421–8432. 10.2147/IJN.S218488 PMC6817714 31749616

[B132] Mukherjee A. Waters A. K. Kalyan P. Achrol A. S. Kesari S. Yenugonda V. M. (2019). Lipid-polymer hybrid nanoparticles as a next-generation drug delivery platform: state of the art, emerging technologies, and perspectives. Int. J. Nanomed. 14, 1937–1952. 10.2147/IJN.S198353 PMC6430183 30936695

[B133] Mukherjee S. Chowdhury D. Kotcherlakota R. Patra S. Bhadra M. P. Bojja S. (2014). Potential theranostics application of bio-synthesized silver nanoparticles (4-in-1 system). Theranostics 4, 316–335. 10.7150/thno.7819 24505239 PMC3915094

[B134] Mukherjee S. Dasari M. Priyamvada S. Kotcherlakota R. Bollu V. S. Patra C. R. (2015). A green chemistry approach for the synthesis of gold nanoconjugates that induce the inhibition of cancer cell proliferation through induction of oxidative stress and their *in vivo* toxicity study. J. Mater. Chem. B. 3, 3820–3830. 10.1039/c5tb00244c 32262856

[B135] Mukherjee S. Patra C. R. (2017). Biologically synthesized metal nanoparticles: recent advancement and future perspectives in cancer theranostics. Future Sci. OA. 3, FSO203. 10.4155/fsoa-2017-0035 28884002 PMC5583654

[B136] Mukherjee S. Sau S. Madhuri D. Bollu V. S. Madhusudana K. Sreedhar B. (2016). Green synthesis and Characterization of monodispersed gold nanoparticles: toxicity study, delivery of doxorubicin and its bio-distribution in mouse model. J. Biomed. Nanotechnol. 12, 165–181. 10.1166/jbn.2016.2141 27301182

[B137] Musette P. Bouaziz J. D. (2018). B cell modulation strategies in autoimmune diseases: new concepts. Front. Immunol. 9, 622. 10.3389/fimmu.2018.00622 29706952 PMC5908887

[B138] Nguyen T. L. Choi Y. Kim J. (2019). Mesoporous silica as a versatile platform for cancer immunotherapy. Adv. Mater. 31, e1803953. 10.1002/adma.201803953 30417454

[B139] Nishiguchi A. Taguchi T. (2020). Sustained-immunostimulatory nanocellulose scaffold to enhance vaccine efficacy. J. Biomed. Mater. Res. 108, 1159–1170. 10.1002/jbm.a.36890 31990447

[B140] Palucka K. Banchereau J. (1999). Dendritic cells: a link between innate and adaptive immunity. J. Clin. Immunol. 19, 12–25. 10080101 10.1023/a:1020558317162

[B141] Pancer Z. Cooper M. D. (2006). The evolution of adaptive immunity. Annu. Rev. Immunol. 24, 497–518. 10.1146/annurev.immunol.24.021605.090542 16551257

[B142] Parkin J. Cohen B. (2001). An overview of the immune system. Lancet 357, 1777–1789. 10.1016/S0140-6736(00)04904-7 11403834

[B143] Pasare C. Medzhitov R. (2004). Toll-like receptors: linking innate and adaptive immunity. Microb. Infect. 6, 1382–1387. 10.1016/j.micinf.2004.08.018 15596124

[B144] Pattan G. Kaul G. (2014). Health hazards associated with nanomaterials. Toxicol. Ind. Health 30, 499–519. 10.1177/0748233712459900 23012342

[B145] Pauken K. E. Dougan M. Rose N. R. Lichtman A. H. Sharpe A. H. (2019). Adverse events following cancer immunotherapy: obstacles and opportunities. Trends Immunol. 06, 511–523. 10.1016/j.it.2019.04.002 PMC6527345 31053497

[B146] Pearce A. Haas M. Viney R. Pearson S. A. Haywood P. Brown C. (2017). Incidence and severity of self-reported chemotherapy side effects in routine care: a prospective cohort study. PLoS One 12, e0184360. 10.1371/journal.pone.0184360 29016607 PMC5634543

[B147] Peng J. Hamanishi J. Matsumura N. Abiko K. Murat K. Baba T. (2015). Chemotherapy induces programmed cell death-ligand 1 overexpression via the nuclear factor-κb to Foster an immunosuppressive tumor microenvironment in ovarian cancer. Cancer Res. 75, 5034–5045. 10.1158/0008-5472.CAN-14-3098 26573793

[B148] Perez C. R. De Palma M. (2019). Engineering dendritic cell vaccines to improve cancer immunotherapy. Nat. Commun. 11 (10), 5408. 10.1038/s41467-019-13368-y PMC6881351 31776331

[B149] Pusuluri A. Wu D. Mitragotri S. (2019). Immunological consequences of chemotherapy: single drugs, combination therapies and nanoparticle-based treatments. J. Control Release. 7, 130–154. 10.1016/j.jconrel.2019.04.020 31004668

[B150] Qin W. Huang G. Chen Z. Zhang Y. (2017). Nanomaterials in targeting cancer stem cells for cancer therapy. Front. Pharmacol. 8, 1. 10.3389/fphar.2017.00001 28149278 PMC5241315

[B151] Restifo N. P. Dudley M. E. Rosenberg S. A. (2012). Adoptive immunotherapy for cancer: harnessing the T cell response. Nat. Rev. Immunol. 12, 269–281. 10.1038/nri3191 22437939 PMC6292222

[B152] Ribas A. Wolchok J. D. (2018). Cancer immunotherapy using checkpoint blockade. Science. 3, 1350–1355. 10.1126/science.aar4060 PMC7391259 29567705

[B153] Rodallec A. Sicard G. Fanciullino R. Benzekry S. Lacarelle B. Milano G. (2018). Turning cold tumors into hot tumors: harnessing the potential of tumor immunity using nanoparticles. Expet Opin. Drug Metabol. Toxicol. 14, 1139–1147. 10.1080/17425255.2018.1540588 30354685

[B154] Roy Chowdhury M. Schumann C. Bhakta-Guha D. Guha G. (2016). Cancer nanotheranostics: strategies, promises and impediments. Biomed. Pharmacother. 84, 291–304. 10.1016/j.biopha.2016.09.035 27665475

[B155] Ruter J. Barnett B. G. Kryczek I. Brumlik M. J. Daniel B. J. Coukos G. (2009). Altering regulatory T cell function in cancer immunotherapy: a novel means to boost the efficacy of cancer vaccines. Front. Biosci. 14, 1761–1770. 10.2741/3338 19273160

[B156] Sabado R. L. Balan S. Bhardwaj N. (2017). Dendritic cell-based immunotherapy. Cell Res. 27, 74–95. 10.1038/cr.2016.157 28025976 PMC5223236

[B157] Samanta K. Setua S. Kumari S. Jaggi M. Yallapu M. M. Chauhan S. C. (2019). Gemcitabine combination nano therapies for pancreatic cancer. Pharmaceutics. 11. 10.3390/pharmaceutics11110574 PMC6920852 31689930

[B158] Sancey L. Kotb S. Truillet C. Appaix F. Marais A. Thomas E. (2015). Long-term *in vivo* clearance of gadolinium-based AGuIX nanoparticles and their biocompatibility after systemic injection. ACS Nano. 9, 2477–2488. 10.1021/acsnano.5b00552 25703068

[B159] Sancey L. Lux F. Kotb S. Roux S. Dufort S. Bianchi A. (2014). The use of theranostic gadolinium-based nanoprobes to improve radiotherapy efficacy. Br. J. Radiol. 87, 20140134. 10.1259/bjr.20140134 24990037 PMC4453146

[B160] Sarkar S. Alam M. A. Shaw J. Dasgupta A. K. (2013). Drug delivery using platelet cancer cell interaction. Pharm Res. 30, 2785–2794. 10.1007/s11095-013-1097-1 23739991

[B161] Sau S. Alzhrani R. Bhise K. Alsaab H. O. Kashaw S. K. Iyer A. K. (2019). Nanomaterials for tumor immunomodulation and overcoming current clinical challenges. Nanomedicine. 06 (14), 1515–1519 10.2217/nnm-2019-0109 31232169

[B162] Sayes C. M. Liang F. Hudson J. L. Mendez J. Guo W. Beach J. M. (2006). Functionalization density dependence of single-walled carbon nanotubes cytotoxicity *in vitro* . Toxicol. Lett. 161, 135–142. 10.1016/j.toxlet.2005.08.011 16229976

[B163] Schön M. P. Erpenbeck L. (2018). The Interleukin-23/Interleukin-17 Axis links adaptive and innate immunity in psoriasis. Front. Immunol. 9, 1323 29963046 10.3389/fimmu.2018.01323 PMC6013559

[B164] Schreiber R. D. Old L. J. Smyth M. J. (2011). Cancer immunoediting: integrating immunity's roles in cancer suppression and promotion. Science 331, 1565–1570. 10.1126/science.1203486 21436444

[B165] Shankaran V. Ikeda H. Bruce A. T. White J. M. Swanson P. E. Old L. J. (2001). IFNgamma and lymphocytes prevent primary tumour development and shape tumour immunogenicity. Nature 410, 1107–1111. 10.1038/35074122 11323675

[B166] Shi Y. van der Meel R. Chen X. Lammers T. (2020). The EPR effect and beyond: strategies to improve tumor targeting and cancer nanomedicine treatment efficacy. Theranostics 10, 7921–7924. 10.7150/thno.49577 32685029 PMC7359085

[B167] Shukla S. Steinmetz N. F. (2016). Emerging nanotechnologies for cancer immunotherapy. Exp. Biol. Med. 241, 1116–1126. 10.1177/1535370216647123 PMC4950359 27190253

[B168] Simpson J. D. Smith S. A. Thurecht K. J. Such G. (2019). Engineered polymeric materials for biological applications: overcoming challenges of the bio-nano Interface Polymers 11 (9), 1441. 10.3390/polym11091441 PMC6780590 31480780

[B169] Singh A. Peppas N. A. (2014). Hydrogels and scaffolds for immunomodulation. Adv Mater Weinheim 26, 6530–6541. 10.1002/adma.201402105 PMC4269549 25155610

[B170] Singh M. R. (2018). Application of metallic nanomaterials in nanomedicine. Adv. Exp. Med. Biol. 1052, 83–102. 10.1007/978-981-10-7572-8_8 29785483

[B171] Smalley Rumfield C. Pellom S. T. Morillon Y. M. Schlom J. Jochems C. (2020). Immunomodulation to enhance the efficacy of an HPV therapeutic vaccine. J. Immunother. Cancer 6, 8. 10.1136/jitc-2020-000612 PMC7304848 32554612

[B172] Smyth M. J. Dunn G. P. Schreiber R. D. (2006). Cancer immunosurveillance and immunoediting: the roles of immunity in suppressing tumor development and shaping tumor immunogenicity. Adv. Immunol. 90, 1–50. 10.1016/S0065-2776(06)90001-7 16730260

[B173] Song F. Li X. Wang Q. Liao L. Zhang C. (2015). Nanocomposite hydrogels and their applications in drug delivery and tissue engineering. J. Biomed. Nanotechnol. 11, 40–52. 10.1166/jbn.2015.1962 26301299

[B174] Song S. Jin X. Zhang L. Zhao C. Ding Y. Ang Q. (2019). PEGylated and CD47-conjugated nanoellipsoidal artificial antigen-presenting cells minimize phagocytosis and augment anti-tumor T-cell responses. Int. J. Nanomed. 14, 2465–2483. 10.2147/IJN.S195828 PMC6459144 31040669

[B175] Suk J. S. Xu Q. Kim N. Hanes J. Ensign L. M. (2016). PEGylation as a strategy for improving nanoparticle-based drug and gene delivery. Adv. Drug Deliv. Rev. 99, 28–51. 10.1016/j.addr.2015.09.012 26456916 PMC4798869

[B176] Sun J. C. Lanier L. L. (2009). Natural killer cells remember: an evolutionary bridge between innate and adaptive immunity?. Eur. J. Immunol. 39, 2059–2064. 10.1002/eji.200939435 19637199 PMC2819266

[B177] Sun T. Zhang Y. S. Pang B. Hyun D. C. Yang M. Xia Y. (2014). Engineered nanoparticles for drug delivery in cancer therapy. Angew Chem. Int. Ed. Engl. 53, 12320–12364. 10.1002/anie.201403036 25294565

[B178] Sun W. Li S. Tang G. Luo Y. Ma S. Sun S. (2019). Recent progress of nanoscale metal-organic frameworks in cancer theranostics and the challenges of their clinical application. Int. J. Nanomed. 14, 10195–10207. 10.2147/IJN.S230524 PMC6997222 32099352

[B179] Swann J. B. Smyth M. J. (2007). Immune surveillance of tumors. J. Clin. Invest. 117, 1137–1146. 10.1172/JCI31405 17476343 PMC1857231

[B180] Sykes E. A. Dai Q. Sarsons C. D. Chen J. Rocheleau J. V. Hwang D. M. (2016). Tailoring nanoparticle designs to target cancer based on tumor pathophysiology. Proc. Natl. Acad. Sci. USA 113, E1142–E1151. 10.1073/pnas.1521265113 26884153 PMC4780626

[B181] Symowski C. Voehringer D. (2017). Interactions between innate lymphoid cells and cells of the innate and adaptive immune system. Front. Immunol. 8, 1422. 10.3389/fimmu.2017.01422 29163497 PMC5670097

[B182] Takeuchi O. Akira S. (2010). Pattern recognition receptors and inflammation. Cell 140, 805–820. 10.1016/j.cell.2010.01.022 20303872

[B183] Tan J. Saltzman W. M. (2004). Biomaterials with hierarchically defined micro- and nanoscale structure. Biomaterials. 25, 3593–3601. 10.1016/j.biomaterials.2003.10.034 15020133

[B184] Tan P. He L. Han G. Zhou Y. (2017). Optogenetic immunomodulation: shedding light on antitumor immunity. Trends Biotechnol. 35, 215–226. 10.1016/j.tibtech.2016.09.002 27692897 PMC5316489

[B185] Tan Y. Y. Yap P. K. Xin Lim G. L. Mehta M. Chan Y. Ng S. W. (2020). Perspectives and advancements in the design of nanomaterials for targeted cancer theranostics. Chem. Biol. Interact. 329, 109221. 10.1016/j.cbi.2020.109221 32768398

[B186] Tang W. Fan W. Lau J. Deng L. Shen Z. Chen X. (2019). Emerging blood-brain-barrier-crossing nanotechnology for brain cancer theranostics. Chem. Soc. Rev. 48, 2967–3014. 10.1039/c8cs00805a 31089607

[B187] Tay M. Z. Poh C. M. Rénia L. MacAry P. A. Ng L. F. P. (2020). The trinity of COVID-19: immunity, inflammation and intervention. Nat. Rev. Immunol. 06, 363–374. 10.1038/s41577-020-0311-8 PMC7187672 32346093

[B188] Teradal N. L. Jelinek R. (2017). Carbon nanomaterials in biological studies and biomedicine. Adv. Healthc. Mater. 6, 6. 10.1002/adhm.201700574 28777502

[B189] Tian F. Cui D. Schwarz H. Estrada G. G. Kobayashi H. (2006). Cytotoxicity of single-wall carbon nanotubes on human fibroblasts. Toxicol. In Vitro. 20, 1202–1212. 10.1016/j.tiv.2006.03.008 16697548

[B190] Tomar N. De R. K. (2014). A brief outline of the immune system. Methods Mol. Biol. 1184, 3–12. 10.1007/978-1-4939-1115-8_1 25048116

[B191] Tran T. H. Mattheolabakis G. Aldawsari H. Amiji M. (2015). Exosomes as nanocarriers for immunotherapy of cancer and inflammatory diseases. Clin. Immunol. 160, 46–58. 10.1016/j.clim.2015.03.021 25842185

[B192] Turjeman K. Bavli Y. Kizelsztein P. Schilt Y. Allon N. Katzir T. B. (2015). Nano-drugs based on nano sterically stabilized liposomes for the treatment of inflammatory neurodegenerative diseases. PLoS One. 10, e0130442. 10.1371/journal.pone.0130442 26147975 PMC4492950

[B193] Turvey S. E. Broide D. H. (2010). Innate immunity. J. Allergy Clin. Immunol. 125, S24–S32. 10.1016/j.jaci.2009.07.016 19932920 PMC2832725

[B194] Valenta R. Mittermann I. Werfel T. Garn H. Renz H. (2009). Linking allergy to autoimmune disease. Trends Immunol. 30, 109–116. 10.1016/j.it.2008.12.004 19231288

[B195] Vieira D. B. Gamarra L. F. (2016). Getting into the brain: liposome-based strategies for effective drug delivery across the blood-brain barrier. Int. J. Nanomed. 11, 5381–5414. 10.2147/IJN.S117210 PMC5077137 27799765

[B196] Waldhauer I. Steinle A. (2008). NK cells and cancer immunosurveillance. Oncogene. 27, 5932–5943. 10.1038/onc.2008.267 18836474

[B197] Wang H. Franco F. Ho P. C. (2017). Metabolic regulation of Tregs in cancer: opportunities for immunotherapy. Trends Cancer 3, 583–592. 10.1016/j.trecan.2017.06.005 28780935

[B198] Wang J. Sun P. Bao Y. Liu J. An L. (2011). Cytotoxicity of single-walled carbon nanotubes on PC12 cells. Toxicol. In Vitro. 25, 242–250. 10.1016/j.tiv.2010.11.010 21094249

[B199] Wang X. Wang Y. Chen Z. G. Shin D. M. (2009). Advances of cancer therapy by nanotechnology. Cancer Res. Treat. 41, 1–11. 10.4143/crt.2009.41.1.1 19688065 PMC2699095

[B200] Weber J. S. Yang J. C. Atkins M. B. Disis M. L. (2015). Toxicities of immunotherapy for the practitioner. J. Clin. Oncol. 33, 2092–2099. 10.1200/JCO.2014.60.0379 25918278 PMC4881375

[B201] Wertel I. Polak G. Barczyński B. Kotarski J. (2007). Subpopulations of peripheral blood dendritic cells during chemotherapy of ovarian cancer. Ginekol. Pol. 78, 768–771. 18200966

[B202] Whiteside T. L. (2008). The tumor microenvironment and its role in promoting tumor growth. Oncogene 27, 5904–5912. 10.1038/onc.2008.271 18836471 PMC3689267

[B203] Wing E. J. Remington J. S. (1977). Cell-mediated immunity and its role in resistance to infection. West. J. Med. 126, 14–31. 318786 PMC1237425

[B204] Xin Y. Huang M. Guo W. W. Huang Q. Zhang L. Z. Jiang G. (2017). Nano-based delivery of RNAi in cancer therapy. Mol Cancer. 16, 134. 10.1186/s12943-017-0683-y 28754120 PMC5534073

[B205] Xu B. Cui Y. Wang W. Li S. Lyu C. Wang S. (2020). Immunomodulation-enhanced nanozyme-based tumor Catalytic therapy. Adv Mater. 32, e2003563. 10.1002/adma.202003563 32627937

[B206] Xu C. M. Tang M. Feng J. Xia H. F. Wu L. L. Pang D. W. (2020). A liquid biopsy-guided drug release system for cancer theranostics: integrating rapid circulating tumor cell detection and precision tumor therapy. Lab Chip. 04, 1418–1425. 10.1039/D0LC00149J 32195515

[B207] Xu W. Atkins M. B. McDermott D. F. (2020). Checkpoint inhibitor immunotherapy in kidney cancer. Nat. Rev. Urol. 3, 137–150. 10.1038/s41585-020-0282-3 32020040

[B208] Yang G. Liu Y. Wang H. Wilson R. Hui Y. Yu L. (2019). Bioinspired core-shell nanoparticles for hydrophobic drug delivery. Angew Chem. Int. Ed. Engl. 58, 14357–14364. 10.1002/anie.201908357 31364258

[B209] Yang J. Zhang C. (2020). Regulation of cancer-immunity cycle and tumor microenvironment by nanobiomaterials to enhance tumor immunotherapy. Wiley Interdiscip. Rev. Nanomed. Nanobiotechnol. 12, e1612. 32114718 10.1002/wnan.1612

[B210] Yoshioka Y. Kuroda E. Hirai T. Tsutsumi Y. Ishii K. J. (2017). Allergic responses induced by the immunomodulatory effects of nanomaterials upon skin exposure. Front. Immunol. 8, 169. 10.3389/fimmu.2017.00169 28261221 PMC5311046

[B211] You Y. Wang N. He L. Shi C. Zhang D. Liu Y. (2019). Designing dual-functionalized carbon nanotubes with high blood-brain-barrier permeability for precise orthotopic glioma therapy. Dalton Trans. 48, 1569–1573. 10.1039/c8dt03948h 30499579

[B212] Yu W. Sun J. Liu F. Yu S. Hu J. Zhao Y. (2020). Treating immunologically cold tumors by precise cancer photoimmunotherapy with an extendable nanoplatform. ACS Appl. Mater. Interfaces 12, 40002–40012. 10.1021/acsami.0c09469 32805869

[B213] Zacharski L. R. Sukhatme V. P. (2005). Coley's toxin revisited: immunotherapy or plasminogen activator therapy of cancer?. J. Thromb. Haemostasis 3, 424–427. 10.1111/j.1538-7836.2005.01110.x 15748226

[B214] Zhai M. Xu Y. Zhou B. Jing W. (2018). Keratin-chitosan/n-ZnO nanocomposite hydrogel for antimicrobial treatment of burn wound healing: Characterization and biomedical application. J. Photochem. Photobiol. B. 180, 253–258. 10.1016/j.jphotobiol.2018.02.018 29476966

[B215] Zhang Y. Cui Z. Kong H. Xia K. Pan L. Li J. (2016). One-shot immunomodulatory nanodiamond agents for cancer immunotherapy. Adv. Mater. Weinheim 28, 2699–2708. 10.1002/adma.201506232 26833992

[B216] Zhang Y. Xu Y. Li Z. Chen T. Lantz S. M. Howard P. C. (2011). Mechanistic toxicity evaluation of uncoated and PEGylated single-walled carbon nanotubes in neuronal PC12 cells. ACS Nano 5, 7020–7033. 10.1021/nn2016259 21866971

[B217] Zhang Y. Zhang L. Hu Y. Jiang K. Li Z. Lin Y. Z. (2018). Cell-permeable NF-κB inhibitor-conjugated liposomes for treatment of glioma. J. Control Release 11 (289), 102–113. 10.1016/j.jconrel.2018.09.016 30243823

