# Supplementary material for: Nanomaterials-Mediated Immunomodulation for Cancer Therapeutics
Source: Front Chem. 2021 Feb 23;9:629635. doi: 10.3389/fchem.2021.629635 (PMC7940769; doi:10.3389/fchem.2021.629635)
Supplement: Supplementary file 1 [file Table1.docx]

Supplementary Material

Table 1. Nanomaterial-mediated immunomodulation for cancer therapeutics.

| **Nanomaterial** | **Description and Features** | **Mechanism of action** | ***In vivo* model** | **Reference** |
| --- | --- | --- | --- | --- |
| Ferumoxytol (ultrasmall superparamagnetic iron oxide (USPIO) nanoparticles) | NPs with an iron oxide core and outer shell of carboxymethyl dextran  Spherical; 30 nm (approx.) | - Enhanced macrophage recruitment. - Increased production of ROS by macrophages resulting in higher cancer cell cytotoxicity via M1 macrophage polarization. - Increased caspase-3 activity. - Inhibition of growth in subcutaneous adenocarcinomas limited by ferumoxytol metabolism. - Increased populations of CD80^+^ M1 in tumor tissues. - Prevention of metastasis. | - MMTV-PyMT cancer cells and RAW264.7 macrophage cells - NOD.Cg-Prkdc^scid^ Il2rg^tm1Wjl^/SzJ mice tumor models | [1] |
| Polyethylenimine (PEI) coated SPIONs  [PMag NPs] | Spherical; 139 nm-228 nm (approx.) with a ~10 nm core. | - Macrophage activation via TLR4 and ROS (reactive oxygen species) signaling. (MI-like phenotype) - Increased IL-12 secretion - Phosphorylation of p38 MAPK, p44/p42 MAPK, and JNK. - Upregulation of activation markers (CD40, CD80, CD86, and I-A/I-E) | - Murine macrophage-like RAW264.7 cells and THP1 cells. - Female C57BL/6J mice. | [2] |
| Polyhydroxylated fullerenols [C_60_(OH)_22_ and Gd@C_82_(OH)_22_ NPs] | Aggregates; Gd@C_82_(OH)_22_ NPs: 122.34 ± 10.23 nm;  C_60_(OH)_22_ NPs :179.84 ± 18.45 nm | - NP mediated macrophage activation. - Inhibition of cancer cell growth by activated macrophages. - Activated macrophages also inhibit tumor metastasis to the lung. - Enhanced secretion of pro-inflammatory cytokines (IL-1β, IL-6, and TNF-α). - NF-κB-mediated release of multiple cytokines. - Gd@C_82_(OH)_22_ NPs displayed a stronger immunomodulatory effect on the function of macrophages than C_60_(OH)_22_ NPs. | - Murine melanoma (B16) - Murine breast cancer (4T1) Human breast - cancer (MDA-MB-231) - Human lung cancer (A549) - Female BALB/c mice | [3] |
| Gadolinium metallofullerenol NPs  [f-NPs] | Spherical/Ellipsoidal; ~100 nm (approx.)  Surface molecules: Hydroxyl groups. | - The NPs inhibited the production of matrix metalloproteinase (MMP) enzymes under *in vitro* conditions. - The nanomaterial-treated animals displayed isolation of cancer cells within a fibrous collagen cage in various cancer models. - Suppression of tumor metastasis in tissue invasion models. - Significant suppression in the establishment of tumor foci in the lungs. | - MDA-MB-231 cells - MDA-MB-231-Luc cells (constitutively expressing luciferase) - Athymic BALB/c nu/nu female mice. | [4] |
| Biomimetic magnetosome  [ Pa-M/Ti-NCs ] | Core composed of Fe_3_O_4_ magnetic nanocluster (NC) and outer shell of the pre-engineered leukocyte membrane.  Spherical nanoclusters consisting of stacked ∼10 nm Fe_3_O_4_ building units.  Surface molecules: PD-1 antibody (Pa) molecules. | - Long circulation time due to membrane coating. - The Fe_3_O_4_ magnetic nanocluster (NC) core enables magnetic targeting with the help of magnetic resonance imaging (MRI) guidance. - Nanomaterial treatment yielded significantly higher populations of CD4^+^, CD8^+,^ and M1 cells within the tumor microenvironment. - Induced immunological memory responses via effector memory T cells capable of completely suppressing tumor recurrence and increasing survival rate. - Complete suppression of primary tumors along with inhibition of tumor metastasis. | - Mouse leukocyte J774A.1 cell line. - B16F10 cell line. - 4T1 mouse breast cancer cell line. - BALB/c female mice. - C57BL/6 female mice | [5] |
| Silver (AgNPs) and gold nanoparticles (AuNPs) synthesized by fungus *B. tetramera* | AgNPs: Irregular shaped; 4.78 nm 73.49 nm.  AuNPs: Spherical/ hexagonal; 58.4nm and 261.73 nm, respectively. | - AgNPs display antibacterial and antifungal properties while AuNPs exhibit only moderate antibacterial and no antifungal properties. - Generation of reactive oxygen species (ROS) in macrophage cell lines treated with NPs, indicating the ability to stimulate macrophage activation. - Potential to act as an immunomodulatory nano-carrier for drug delivery. | - Mouse macrophage cell line (J774). - Human macrophage cell line (THP1 α). | [6] |
| Poly(lactic-co-glycolic acid) based antigen capturing NPs  [PLGA-AC-NPs] | Surface modified to enable binding of tumor-derived protein antigens (TDPAs)  Spherical; ∼150nm (Before antigen-capture) | - NPs show migration and accumulation in draining lymph nodes (DLNs). - Increased population of CD8^+^ cytotoxic T cells. - Enhanced both CD4^+^ T/Treg and CD8^+^ T/Treg ratios (Treg, regulatory T cells). - Enhanced antigen (TDPAs) presentation to APCs leading to a more robust CD8^+^ T cell activation. - NPs capable of eliciting strong antitumor immune responses by capturing tumor-specific neoantigens and several damage-associated molecular pattern proteins (DAMPs), histone proteins, and alarmins (including HMGB1). | - B16-F10 murine melanoma cell line. - 4T1 mouse breast cancer cell line. - B16F10 melanoma model | [7] |
| Exosome-mimetic nanovesicles derived from M1 macrophages  [M1NVs] | Spherical nanovesicles; ∼189.7 ± 2.5 nm | - Higher cellular uptake of nanovesicles by M2 macrophages. - Repolarization of M2 tumor-associated macrophages (TAMs) to M1 macrophages (*In vitro* and *in vivo*). - Secretion of pro-inflammatory cytokines (IL-6) to build antitumor responses. - Reduced the secretion of anti-inflammatory cytokine (IL–4). - Upregulation of miR-155, miR-125, and miR-21 expression and downregulation of miR-34a, let-7c, and let7f. - Inhibits cancer cell proliferation by M1 macrophage-secreted pro-inflammatory cytokines. - Reduced expression of CCL–18, a metastatic factor, in M2 macrophages. - Accumulation of M1NVs at the tumor site and tumor growth inhibition in the animal model. | - RAW264.7 murine macrophage cell line. - CT26 mouse colon carcinoma cell line. - CT26 colon carcinoma-bearing BALB/c mice | [8] |
| Polystyrene nanoparticles functionalized with carboxyl and amino groups.  [(PS-COOH) and (PS-NH_2_)] | Spherical; (PS-COOH) NPs: 119 ±  19 nm and (PS-NH_2_) NPs: 117 ± 17 nm.  Surface groups: Carboxyl and amino groups. | - NP treatment inhibited M2 polarization of macrophages. - Inhibited the expression of scavenger receptor CD163 and CD200R in M2 macrophages. - Inhibited secretion of IL-10. - NP treatment affected the proliferation of THP-1 but does not induce proliferation of polarized M1 and M2 macrophages. - Potential to be applied as a tool for reprogramming the M1/M2 polarization. - NPS stimulated the expression of TGF-β1 in M1 and M2 macrophages. | THP-1 human monocyte cells were differentiated into MI and M2 macrophages | [9] |
| Amorphous silica (Si) and superparamagnetic iron oxide (SPIO) based engineered nanoparticles (ENPs)  [Si-ENPs and SPIO-ENPs] | Spherical; SPIO-ENPs: ∼13nm and  Si-ENPS: ∼50nm. | - SPIO ENP treated macrophages displayed impaired ability to transition from M1 to M2, suppressed IL-10 secretion, and enhanced TNFα production. - Modulation of TLR4-mediated gene regulation by both NPs. - Reduction in phagocytic activity of macrophages. | Wildtype and SR-A deficient mice bred on a C57/BL6 background | [10] |
| Superparamagnetic iron oxide nanoparticles  [SPIONs] | Commercially available Resovist™ having a core consisting of Fe_3_O_4_ (magnetite) and Fe_2_O_3_ (maghemite) and an outer shell made up of carboxy-dextran.  Spherical core-shell; ∼80nm. | - Carboxy-dextran coating enhances the uptake of SPIONs by macrophages. - Upregulation of intracellular ferritin and cathepsin L levels in macrophages. - SPION primes THP1 derived M2 macrophages towards a high CD86^+^ and - high TNF α + macrophage subtype | M2 macrophages derived from human monocyte (THP1) cells | [11] |
| Differentially charged SPIONs  [S+ (Positive), S- (Negative), SN(Neutral)] | Commercially available Ferumoxytol (Feraheme®) based SPIONs  Nano-aggregates within size range: 15–25 nm  S+ SPIONs: 19.4 ± 0.8 nm,  SN SPIONs: 15.9 ± 0.2 nm, and  S- SPIONs: 21.3 ± 1.6 nm, | - Cellular uptake efficiency: S+> S-> SN. - Macrophage repolarization effect (M2-like to M1-like phenotype): - S− > S+> SN. - SN induced expression of TNF-α and decrease expression of IL-10 and VEGF. - Significant enhancement in the Fenton effect in cancer cells along with a higher expression of caspase 9. - S- and S+ lead to higher CD11b and CD80 expression lower expression of CD206. - S- and S+ pre-treated macrophages displayed the highest anti-tumor response by causing tumor retardation *in vivo* while SN pre-treated group showed no anti-tumor efficacy. | - HT1080 human fibrosarcoma cell line. - RAW264.7 macrophage cell line. - BALB/c normal mice and nude mice | [12] |
| A ferrimagnetic vortex-domain iron oxide nanoring and graphene oxide hybrid nanoparticle  [FVIOs-GO] | A magneto-thermodynamic (MTD) therapy combining the magnetothermal effect and the reactive oxygen species (ROS)-related immunologic effect.  FVIOs-GO acts as an MTD agent.  Ring-shaped structure attached to GO nanosheets; ∼166.6 nm.  Surface molecules: Cys-Arg-Glu-Lys-Ala (CREKA) | - FVIOs-GO NPs displayed high thermal conversion efficiency. - Generated amplified ROS level under an alternating magnetic field (AMF) both *in vitro* and *in vivo.* - Immunogenic cell death of 4TI cells. - Promoted macrophage polarization from the M2 phenotype to pro-inflammatory M1 phenotypes. - Elevated levels of tumor-infiltrating T lymphocytes. - Displayed good *in vivo* safety in a healthy Balb/c mice model. - Systemically delivered MTD eliminated the tumor with a low dosage and shorter AMF exposure times. | - Mouse breast cancer (4TI) cell line. - RAW264.7 macrophage cell line. - Mouse Subcutaneous 4T1 breast tumor model. - Female Balb/c mice model | [13] |
| Carbon-based NMs   - Graphite nanofibers (GNF) - Multiwalled carbon nanotube (rCNT) - Multiwalled carbon nanotube (Baytubes) | GNF: Fiber-type; Length: 10,000 nm, Diameter: 140 nm.  rCNT: Tube-type; Length: 13,000 nm, Diameter: 50 nm.  Baytubes: Tube-type; Length: 1,000 nm, Diameter: 15 nm. | - **GNF**: Increased secretion of Interleukin 10 (IL‐10) and Interleukin 1β (IL‐1β), activated pro‐inflammatory and IL‐1β‐related pathways. - **rCNT**: Increased secretion of pro‐inflammatory cytokine IL‐1β as well as anti‐inflammatory cytokine IL‐10, triggered chemotaxis and cytokine‐mediated pathways. NF‐κB activation. - **Baytubes**: Moderate secretion of IL‐10, but no significant secretion of the M1 cytokines IL‐1β or TNF, elicited TGF‐β2 production. NF‐κB activation. | Macrophage‐like THP‐1 cells | [14] |
| Synthetic high-density lipoprotein-like nanoparticles  [HDL NPs] | The HDL NPs were composed of dipalmitoyl-sn-glycero-3 phosphocholine (DPPC) and 1,2 dipalmitoyl-sn-glycero-3-phosphoethanolamine-N-[3-(2-pyridyldithio)propionate] (PDP-PE)  Spherical; ∼7-13 nm | - NPs specifically bind to the scavenger receptor type B-1 (SCARB1) on myeloid-derived suppressor cells (MDSCs) - Inhibition of MDSCs activity. - In animal models, enhancement of adaptive immune responses, inhibition of tumor growth, lower metastatic tumor burden, and enhanced survival. - A higher population of CD8^+^ T cells and reduced Treg cells. - Inhibition of tumor metastasis and increased survival in animal models. | - B16F10 melanoma cell line. - Lewis Lung Carcinoma (LLC) cell line. - C57Bl/6 melanoma metastasis mice models. | [15] |
| Hyaluronic acid (HA)-decorated superparamagnetic iron oxide nanoparticles (IONs)  [HIONs] | Macrophages were artificially reprogrammed with HA-coated IONs  (HION@Macs) | - Robust and long-term activation of macrophages due to HA. - M2 to M1 polarization of macrophages. - Intracellular iron ions activated NF-kB and initiated a TNF-α associated pro-inflammatory macrophage response. - Enhance tumor targeting and prolonged intratumoral retention via magnet guided location and retention. - Provided resistance against the immunosuppressive tumor micro-environment. - Reprogrammed macrophages (HION@Macs) displayed increased generation of bioactive components such as reactive oxygen species, bioactive cytokines) leading to effective tumor growth suppression. | - RAW 264.7, murine macrophage cell line. - 4T1 mouse mammary carcinoma cell line. - 4T1 mice breast tumor model with BALB/c background | [16] |
| Gold nanoparticles (AuNPs) conjugated to ovalbumin antigen (OVA)  [OVA-AuNps] | Spherical NPs of various sizes.  5, 15, 50 and100 nm.  Surface molecules: Ovalbumin (OVA) | - The 50−100 nm OVA-AuNPs showed greater retention than the 5−15 nm NPs after 48 h of injection. - 5-15 nm NPs entered follicular dendritic cells (FDCs) but were cleared out in 48h while 50-100nm remained on the surface for over 5 weeks. - Differential binding of NPs to the surface due to greater adsorption of complement C3. - The size of the antigen conjugated nanocarrier governed the intra-lymph node transport and specific cell interactions. - The 50−100 nm-sized OVA-AuNPs displayed enhanced antigen presentation, increased humoral immune responses of germinal center B cell formation, and higher production of antigen-specific antibodies. - NPs capable of tuning humoral immunity based on their sizes. | - C57BL/6 mice - Genetically modified female C57BL/6 C3, Cr2, and CD19 knockout mice. | [17] |
| Cuttlefish ink derived nanoparticles  [CINPs] | NPs rich in melanin and contained a variety of amino acids and monosaccharides.  Spherical with rugged surface; ∼100 nm. | - Activated mitogen-activated protein kinase (MAPK) signaling pathway. - Repolarization of TAMS from M2-like phenotype to antitumor M1-like phenotype. - Exhibited high photothermal effect and tumor cell killing ability under near-infrared (NIR) irradiation. - A potential candidate for photothermal therapy. - Reduced tumor growth and metastasis. - Enhanced recruitment of cytotoxic T lymphocytes to the tumor site. | - Colon adenocarcinoma cell line (CT26). - RAW264.7 macrophage cell line. - Female BALB/c mice. - CT26 colon carcinoma bearing mice. | [18] |
| Ink-blue titanium dioxide (BTiO_2_) modified with chitooligosaccharides (COS)  [BTC NPs] | Irregular-shaped; ∼160 nm | - Inhibition of liver tumor cell proliferation and growth. - Repolarization of TAMS from M2-like phenotype to antitumor M1-like phenotype. - High photothermal conversion efficiency. - Higher tumor inhibition rate in combination therapy. | - RAW264.7 macrophage cell line. - Mouse H22 liver cancer model. | [19] |
| Melanin NPs camouflaged with red blood cell (RBC) membrane.  [Melanin@RBC] | Spherical; ∼241 nm | - Displayed strong absorption at the NIR region and higher photothermal conversion efficiency. - Improved circulation time due to RBC membrane camouflaging and higher accumulation at the tumor site. - Optimal tumor accumulation achieved *in vivo* by photoacoustic imaging. - Higher PTT efficacy than bare melanin nanoparticles *in vivo.* | - Human lung adenocarcinoma cell line (A549). - Human embryonic kidney cell line (HEK-293T) - A549 tumor-bearing mice. - Male Balb/c nude mice. | [20] |
| Gold (Au) and copper-molybdenum sulfide (CMS) based heterostructures.  [Cu_2_ MoS_4_ (CMS)/Au] | 2D CMS sheets embedded with spherical AuNPs;  AuNPs: ∼2 nm  CMS: ∼85.15 nm  (CMS)/Au: ∼106.57 nm | - Enhanced cellular uptake - Higher photothermal conversion property. - Ability to utilize over-expressed H_2_O_2_ within TME to produce O_2_ and alleviate hypoxic conditions. - Enhanced ROS generation leading to tumor cell apoptosis/necrosis. - DCs maturation, cytokine secretion, and a higher population of cytotoxic CD8^+^ T cells and helper CD4^+^ T cells. | - Human cervix cancer cell line (HeLa). - Murine cervical carcinoma cell line (U14) - U14 subcutaneous tumor-bearing mouse model with Balb/c background. | [21] |
| Bionized nanoferrite  (BNF) nanoparticles  [BNF-NPs] | The BNF NPs consisted of a magnetic iron oxide core coated with hydroxyethyl starch (shell) and an amine-functionalized surface.  Core-shell nanostructures; 99 ±3 nm  Surface molecules: Trastuzumab (BH) | - Immune status of the host governed retention of antibody-labeled NPs in tumors - In animal models with an intact immune system, better retention of antibody-labeled NPs within the tumor than unlabelled NPs. - Tumor-associated immune cells internalized antibody-labeled NPs. - CD8^+^ T cell infiltration and tumor growth retardation. | - MDA-MB-231, MCF7/neo, MCF7/HER2, HCC1954 and BT474 cell lines. - RAW264.7 macrophage cell line. - Athymic nude mice with HCC1954 tumors - FVB/N female mice - Transgenic (huHER2) mice (FVB/N background) | [22] |
| Iron oxide and ovalbumin (OVA) nanocomposite  [Fe_3_O_4_-OVA NPs] | Fe_3_O_4_ NPs were surface-functionalized with carboxyl groups to attach OVA.  Nanoaggregates; ∼ 20 - 40 nm  Surface molecules: Tumor antigen model protein, OVA | - Stimulated maturation of bone marrow-derived dendritic cell (BMDCs) - Stimulated activation of T cells and macrophages. - Inhibited subcutaneous and metastatic B16-OVA tumor growth. - Prevented formation of metastatic and subcutaneous tumors. | - Female C57BL/6 mice. - RAW264.7 macrophage cell line. - B16F10 murine melanoma cell line. - OVA-transfected B16 melanoma cell line (B16-OVA). - B16F10 melanoma model. | [23] |
| Intracellularly generated gold NPs from melanoma B16F10 cells and dendritic cells (DCs)  [AuNP@DC_B16F10_] | NPs generated intracellularly in B16F10 cells, isolated and then further internalized by DCs and secreted as DCs derived vesicles.  Core-shell structure; Au NP ∼40 nm  Outer membrane ∼6−8 nm | - Displayed photothermal therapeutic efficacy. - Eradication of primary tumors with laser irradiation. - Exhibited tumor accumulation and enhanced tumor biodistribution. - Migration and accumulation in lymph nodes establishing antitumor immunity. - Exhibited tumor suppression and inhibition of tumor metastasis. - Prevented future tumor relapse when applied with combinatorial therapies. - Significantly improved overall survival of animal models. | - B16F10 murine melanoma cell line. - DC2.4 cell line. | [24] |
| Engineered dendritic cells (DCs) decorated with polydopamine nanostructures via Ca^2+^ bridge.  [DC ^Ca/PDA^] | Polydopamine particle size; Variable within the nano-range.  Surface molecules: Ca^2+^ bridges and polydopamine nanostructures. | - Engineering bone marrow-derived DCs allowed unique bi-directional control over DC maturation. - Polydopamine NMs suppressed DC activation via scavenging of ROS. - Laser irradiation (808 nm) remotely relieved this suppression and promoted DC maturation via photothermal effect. | - DC2.4 cell line. - C57BL/6 mice. | [25] |

Table 2. Nanomaterials (NMs) as targeted delivery systems for immunomodulating agents

| **Nanomaterial** | **Description and Features** | **Cargo**  **molecule(s)** | **Target** | **Immunomodulation** | ***In vivo* model** | **Reference** |
| --- | --- | --- | --- | --- | --- | --- |
| Artificial APCs encapsulating poly(DL-lactide-co-glycolide) nanoparticles  [PLGA-NPs] | Human mature dendritic cells (mDCs) containing PLGA NPs loaded with peptide antigens.  Spherical; 150-500 nm (approx.) | Tumor antigenic peptide cocktail | Human dendritic cells (DCs) | - Enhanced delivery of antigenic peptides into DCs. - Stimulation of a robust tumor antigen-specific cytotoxic T lymphocyte (CTL) response (*in vitro* and *in vivo*). | - Transgenic adenocarcinoma mouse prostate (TRAMP)-C2 cell lines - C57BL/6 mice model | [26] |
| PLGA NPs encapsulating TLR 7/8 bi-specific agonists  [PLGA-TLR7/8 NPs] | TLR7/8 agonist 522 (or 528) loaded PLGA NPs  Spherical; 156 ± 26 nm (approx.) | TLR 7/8 bi-specific agonists 522 (or 528) | Dendritic cells  (DCs) | - Enhanced expression of co-stimulatory molecules - Improved antigen presentation via MHC I by DCs - Migration and accumulation of NPs to draining lymph nodes. - Promotion of DC activation and maturation. - Development of antigen-specific T cell response. - Inhibition of B16F10 tumor growth. | - C57BL/6 mice model - B16F10 lung metastasis mice model. - Subcutaneous melanoma model. - Balb/c mice bearing IR Renca-GL tumors | [27] |
| PLGA NPs encapsulating ovalbumin (OVA) and polyinosinic-polycytidylic acid (poly I:C)  [PLGA-OVA-Poly I:C NPs] | PLGA-NP system encapsulating  both ovalbumin (OVA) and poly I:C ((Toll-like receptor 3 ligand))  Spherical; 200 ± 3.69 nm (approx.) | Model antigen Ovalbumin (OVA) and Toll-like receptor 3 ligand (polyinosinic-polycytidylic acid sodium salt) | Dendritic cells  (DCs) | - Cellular uptake by DCs. - Increase the efficiency of targeted intracellular delivery of cargo. - Promotion of DC maturation. - Antigen presentation via major histocompatibility complex (MHC) class I molecules. - Stimulation of antigen-specific potent antitumor efficacy. - Prolonged survival in tumor-bearing mice. | Female C57BL/6 mice | [28] |
| Cancer cell membrane-coated PLGA nanoparticles  [CCM-PLGA NPs] | Core-shell with PLGA core and cancer cell membrane as the outer shell.  Spherical; 110 nm (approx.)  Surface molecules: Monophosphoryl lipid A | Cancer cell membrane antigens | Antigen-Presenting Cells  (cancer cells-specific affinity) | - Upregulation of the DC maturation markers (CD40, CD80, and CD86) - Stimulation of tumor antigen-specific T-cells. | - Human MDA-MB-435; - B16F10 mouse melanoma cell lines. | [29] |
| Cancer cell membrane-coated PLGA nanoparticles with mannose  [NP-R@M-M NPs] | Core-shell with PLGA core and cancer cell membrane as the outer shell  Spherical; 160 nm (approx.)  Surface molecules: mannose-conjugated 1,2-distearoyl-sn-glycero-3-phosphoethanolamine- N-methoxy  (polyethylene glycol) (DSPE-PEG-Man) | Cancer cell membrane antigens and Toll-like receptor 7 agonist, imiquimod (R837) | Antigen Presenting Cells (APCs) | - Enhanced DC uptake. - *In vitro* secretion of TNF-α and IL-12p40, stimulation of DC maturation. - Displayed *in vivo* migration of NPs to draining lymph nodes. - *In vivo* inhibition of tumor progression. - Stimulation of antigen-specific antitumor immunity. | Murine B16-OVA melanoma tumor model. | [30] |
| Mannose-inserted-RBC-membrane  coated PLGA nanoparticles  [ManRBC-NP_hgp_] | Core-shell with PLGA core and erythrocyte cell membrane as the outer shell  Spherical; 156 nm (approx.)  Surface molecules: Monophosphoryl lipid A; antigenic peptide (hgp100_25-33_); and DSPE-PEG-mannose (DSPE-PEG-Man) | Cancer cell membrane antigens and hgp100_25-33_ | APCs in the lymphatic organs | - Enhanced *in vitro* cellular uptake - Migration and retention in draining lymph nodes. - Tumor growth inhibition *in vivo.* - Suppression of tumor metastasis - Enhanced IFN-γ secretion - Stimulation of CD8^+^ T cell responses. | Subcutaneous xenograft B16F10 melanoma model. | [31] |
| PLGA NPs carrying murine melanoma antigenic peptides and toll-like receptor 4 (TLR4)  [MPLA-PLGA-NPs] | Spherical; 80-27 nm (approx.) | Murine melanoma antigenic peptides (hgp100_25-33_; TRP2_180-188_) and Monophosphoryl lipid A  (MPLA) | Antigen Presenting Cells (APCs) | - Selective uptake of NPs by APCs. - Migration and retention in draining lymph nodes. - NP-mediated antigen presentation on APCs - Antigen-specific T-cell responses. - Enhanced secretion of inflammatory cytokines, TNF-a, and IL-12. - DC activation and maturation. - Partial inhibition of tumor growth. | - B16-F10 murine melanoma cells. - C57BL/6 (B6) mouse models. | [32] |
| T-cell membrane camouflaged Trametinib loaded PLGA NPs  [19LF6- Trametinib PLGA NPs] | Core-shell structure with Trametinib loaded PLGA core and coated with an outer shell of the hybridoma T-cell membrane (19LF6).  Spherical; 193 ± 56 nm (approx.)  Surface molecules: Melanoma-specific anti-gp100/HLA-A2 T-cell receptors (TCR) | FDA-approved melanoma chemotherapeutic drug Trametinib. | gp100 positive melanoma cells | - Higher stability of the coated NPs. - Enhanced cellular uptake in melanoma cell lines. - Controlled/sustained drug release. - Increased tumor retention | - Gp100 posiitve melanoma cell lines DM-6 and 1520. - Subcutaneous xenograft mice model of melanoma | [33] |
| Polyethylenimine (PEI)-coated polymeric PLGA nanoparticles containing ovalbumin (OVA)  [PEI-coated PLGA (OVA) NPs] | PEI-coated PLGA NPs encapsulating OVA  Spherical; 150–250 nm (approx.) | Model antigen-Ovalbumin (OVA) | Dendritic cells  (DCs) | - Targeted entry of NPs into DCs via phagocytosis or macropinocytosis - Enhanced delivery of OVA antigen without inducing DC maturation. - Cross-presentation of antigen MHC class I molecules. - Induction of antigen-specific CTL immune response. | Female C57BL/6 mice | [34] |
| Biodegradable poly(γ -glutamic  acid) (γ –PGA) nanoparticles containing ovalbumin (OVA)  [γ –PGA (OVA) NPs] | Polymeric γ –PGA NPs encapsulating OVA  Spherical; 252 nm (approx.) | Model antigen-Ovalbumin (OVA) | Antigen Presenting Cells (APCs) | - Promotion of DC maturation - Induction of innate and adaptive immune responses via Toll-like receptor 4 (TLR4) and MyD88 signaling pathways | - MyD88-knockout C57BL/6 mice - TLR4-deficient (C3H/HeJ) mice | [35] |
| PLGA Nps co-loaded with pentaerythritol lipid A (PET lipid A) and OVA  [PLGA (OVA+PET lipid A) NPs] | Spherical; 250-600 nm (approx.) | Pentaerythritol lipid A (PET lipid A) [(TLR-4) agonist]  and Ovalbumin OVA | Dendritic cells  (DCs) | - Secretion of cytokine, IL-12p70. - Upregulation of key costimulatory factors CD86 and CD40 in DCs. - DC maturation and T cell activation. - Induction of antigen-specific immunity and enhanced antitumor response. | C57BL/6 mice | [36] |
| Gold nanoparticles (AuNPs) conjugated with antigens or immunomodulators  [NanoAu-Cocktail] | Dendritic cells were pulsed with NanoAu-Cocktail for enhanced DC immunotherapy.  Spherical; 60nm and 80 nm (approx.)  Surface molecules: OVAp or CpG-ODNs | OVA peptides, (OVAp) or immunomodulators (CpG-ODNs)  (agonists of TLR9) | Dendritic cells  (DCs) | - Au-mediated activation of bone-marrow-derived dendritic cells (BMDCs). - Enhanced antigen cross-presentation and maturation pf BMDCs - Promoted homing of intravenously injected DCs to liver-draining lymph nodes via higher expression of CCR7. - Stronger CD8+ T cell immune responses in liver lymph nodes | - Male C57BL/6J mice. - L2G85 (FVB) mice expressing Fluc back-crossed with C57BL/6J mice (L2G85. C57BL/6J mice.) - Fluc+ BMDCs induced from L2G85. | [37] |
| Amphiphilic poly(ethylene glycol) (PEG) polymer-prodrug conjugate of an imidazoquinoline TLR7/8 agonist  [PEG5k-GL2-IMDQ vesicles] | Imidazoquinoline (IMDQ) TLR7/8 agonist conjugated to PEG via hydrophobic enzyme-responsive and self-immolative β-GUS-sensitive linker (GL).  Hollow and spherical self-assembled vesicular NPs; 195 nm (approx.) | Imidazoquinoline (IMDQ) TLR7/8 agonist | Endosomal compartments of APCs | - Migration of NPs to lymph nodes, - DC activation via TLR7/8 pathway - Upregulation of DC maturation markers (CD40, CD80, and CD86). - Prolonged *in vivo* immune stimulation due to sustained release. | Transgenic luciferase reporter mice model (IFNβ+/Δβ-luc) with a BALB/c background | [38] |
| Hybrid vesicle-based nanocarriers co-loaded with doxorubicin (DOX) and gefitinib (GE)  [Hybrid c(RGDm7)-LS-GE/DOX] | Nanocarriers formed by the fusion of exosomes containing CD47 with c(RGDm7)-modified nanocarriers co-loaded with doxorubicin (DOX) and gefitinib (GE)  Double-membrane spherical vesicles; 160 nm (approx.)  Surface molecules: Cationized mannan (M-EV) | Doxorubicin (DOX) and gefitinib (GE) | Macrophages | - M-EV enhanced macrophage targeting. - Minimization MPS uptake - Tumor growth inhibition - Prolonged survival in tumor models - Promotion of apoptosis - Suppression of angiogenesis | - Murine RAW 264.7 macrophages, DC2.4, and A549 cell lines. - Subcutaneous tumor model of A549 lung cancer in BALB/c mice | [39] |
| Polyanhydride (20:80 CPTEG:CPH) nanoparticles  [CPTEG:CPH NPs] | Polyanhydride copolymer of 1,6-bis(p-carboxyphenoxy)hexane (CPH)  1.8-bis(p-carboxyphenoxy)-3,6-dioxaoctante (CPTEG) in ratio 20:80 encapsulating Ova  Spherical; 167± 29.2 nm (approx.) | Ovalbumin OVA | Dendritic cells  (DCs) | - Generation of antigen-specific CD8^+^ T cell memory without overt inflammatory responses. - Activation of CD8α+ of DCs, innate responses, and upregulation of co-stimulatory molecules. - Suppressed tumor progression | - E.G7-OVA and EL4 lymphoma cell lines - Female C57BL/6 mice | [40] |
| Poly(beta-amino ester) (PBAE) nanoparticles encapsulating Cyclic dinucleotides (CDNs)  [CDN PBAE NPs] | CDNs are a potent stimulator of Interferon Receptor (STING) agonist.  Spherical; 100 nm (approx.) | Cyclic dinucleotides (CDNs)  (STING agonist) | Human monocytes | - Improved efficiency of CDN delivery to human monocytes. - Enhanced activation of IRF3 transcription factor via STING. - Development of robust immune response. - Reduction in tumor growth *in vivo.* - No significant loss of efficacy of nanomedicine formulation after lyophilization and long-term storage (>9 months). | - RAW 264.7 murine macrophages, THP1 human monocyte cell line, and B16-F1 melanoma cells - Female C57BL/6 mice with B16 melanoma tumors | [41] |
| Polyethylene glycol (PEG) and polyethyleneimine (PEI) functionalized graphene oxide (GO)  [GO-PEG-PEI NPs] | GO sheets containing covalently conjugated PEI and PEG as a carrier complex for unmethylated cytosine-phosphate-guanine (CpG) oligonucleotides (ODNs)  Dispersed sheet-like structures; thickness of  around 10 nm (approx.) | CpG ODNs | Macrophages | - Toll-like receptor 9 (TLR9) mediated recognition of CpG ODNs. - Enhanced cytokine expressions (IL-6, TNF-α). - High biocompatibility. - Improved efficiency in tumor reduction when combined with photothermal therapy. | - Murine macrophage-like RAW264.7 cells - Subcutaneous tumors in BALB/c mice induced by CT26 colon carcinoma cells | [42] |
| Superparamagnetic iron oxide nanoparticles (SPIONs) coated with recombinant heat shock protein 70 (Hsp70)  [Hsp70-SPIONs] | Spherical; 44.3 ± 3.2 nm (approx.) | Hydrophobic BP3 peptide (KKFYQLALTKK) (BP3) labeled with FITC. | Dendritic cells  (DCs) | - Stimulation of tumor-specific, CD8^+^ cytotoxic T cell response. - Delayed tumor progression *in vivo.* - Prolonged survival - Increased secretion of IFNγ - Enhanced infiltration of memory CD45RO+ and cytotoxic CD8+ T cells within glioma. | Orthotopic C6 glioma rat model. | [43] |
| Nanoliposomes containing AE36 HER2/neu-derived peptide (with CpG ODN)  [DOTAP/DOPE/Chol/AE36+CpG] | Nanoliposomes composed of  N-[1-(2, 3-Dioleoyloxy) propyl]-N, N, N  Trimethylammonium methyl-sulfate (DOTAP), dioleoylphosphatidylethanolamine (DOPE) and cholesterol (DDC) [1:1:1 molar ratio]  Spherical; 251.3-265.8 nm (without CpG)  372.6-455.2 nm (with CpG) | AE36 HER2/neu derived peptide  (Ac-GVGSPYVSRLLGICL-NH2) | Antigen Presenting Cells (APCs) | - Induction of both CD8^+^ and CD4^+^ antigen-specific responses. - Induction of CTL activation and IFN γ secretion - Stimulates the migration of DCs to draining lymph nodes. - Enhanced anti-tumor immunity. - Shrinkage in tumor size and prolonged survival time. - Induction of MAP kinase ERK pathway. | - TUBO cancer cell line. - BALB/c mice model bearing HER2+ breast cancer | [44] |
| Nanoliposomes targeted to Fc receptors (FcRs) containing TLR ligands with multiepitopic  Antigen  [FcRs-TLR NPs] | Spherical; 125.0 ±5.5 to 207.8 ± 36.7 nm (approx.) | Co-encapsulation of several TLR adjuvants along with a mixture of synthetic Luteinizing hormone-releasing hormone (LHRH) peptide antigens | Human dendritic cells  (hDCs) | - Specific cellular uptake - Improved induction of DC activation - Higher cytokine production and lymphocyte activation - Co-encapsulation of three TLR adjuvants shows better immune. - Nanovaccine for treatment of prostate cancer | Human monocytes from peripheral blood mononuclear cells (PBMCs) | [45] |
| Polyethylene glycol (PEG) hydrogel-based subunit vaccine  [PEG-CpG] | Antigen peptide and CpG co-conjugated to NPs via either a disulfide or a thioether linkage.  Elongated rod-like nanostructures; 240-270 nm (approx.) | CSIINFEKL antigen peptide and CpG ODN | Bone marrow-derived dendritic cells (BMDCs) | - Particle internalization by BMDCs - BMDCs activation and maturation. - Stimulation of Tumor-specific CD8+ cytotoxic T lymphocytes (CTLs) - Sustained release of peptide over 72 hours. - Inhibition of EG7-OVA tumor growth | Female C57BL/6J based EG7-OVA mouse tumor model | [46] |
| poly(lactic-coglycolic acid)-b-polyethylene glycol (PLGA-PEG) nanoparticles  [PLGA-PEG-NP] | Spherical; 50nm-90nm (approx.)  Surface molecules: Recombinant human hyaluronidase PH20 (rHuPH20) embedded into the shell. | Doxorubicin | Tumor site | - Conjugated rHuPH20 was more efficient than free rHuPH20 in enhancing NP diffusion into the matrix. - Maintenance of enzyme activity without a decrease in circulation time. - Enhanced tumor penetration and accumulation. - Tumor inhibition and higher percentage of increased life span. | - BALB/c mice bearing 4T1 tumors. - The triple-negative 4T1 syngeneic mouse tumor model. | [47] |
| Liposomal NPs incorporated with STING (Stimulator of IFN genes) activator.  [liposomal-cGAMP-NPs] | STING Activator: Cyclic [G(3′,5′)pA(3′,5′)p] (cGAMP)  Spherical; 85 ± 27 nm (approx.) | cGAMP NPs | Cytosol of bone marrow-derived macrophages  (BMDMs) | - Amplification of innate immune activation. - Higher IFN-I production - Enhanced STING-mediated antitumor response. - Tumor suppression. - Prevention of secondary tumors by eliciting T cell response and establishing antitumor memory. | - Orthotopically transplanted model [C3(1)Tag model] - Aggressive B16F10 subcutaneous melanoma model. - Spontaneous genetic engineered mouse (GEM) model. [C3(1)Tag GEM] | [48] |
| Lyophilized nanoemulsion of Imiquimod (R837) and resiquimod (R848) loaded with TLR7/8 agonists  [NE (TLR7/8a)] | Spherical; NE (R837): 147.1 ±12.9 nm  NE (R848): 146.5 ± 6.2 nm | TLR7/8 agonists | Antigen Presenting Cells (APCs) | - Activation of innate immune cells. - Migration of NPs to lymph nodes and uptake by APCs. - Higher secretion of TNF-α, IFN-α IFN-β, and IL-6. - Polarization of TAMS (Tumor-associated macrophages) towards the M2 phenotype. - Tumor growth inhibition. - Prolonged survival primary as well as re-challenged tumor models. | - Mouse melanoma cell line B16F10, B16F10-OVA and mouse lung epithelial cell line TC-1 - C57BL/6 mice based B16F10, B16F10-OVA, and TC-1 tumor models. | [49] |
| Bioconjugated manganese dioxide nanoparticles (MnO_2_)  [Man-HA-MnO_2_] | MnO_2_ NPs coated with hyaluronic acid and conjugated with mannan.  Spherical; 180nm-200nm (approx.) | Doxorubicin | Tumor-associated macrophages (TAMs) | - Enhanced tumor oxygenation. - Down-regulation of hypoxia-inducible factor-1 α (HIF-1α) and vascular endothelial growth factor (VEGF) in the tumor. - Inhibition of tumor cell proliferation and growth. - Repolarization of M2 macrophages to pro-inflammatory, antitumor M1 macrophages. - Potential use as *T*_1_-weighted MRI contrasting agent for tumor imaging and tumor hypoxia detection | - RAW264.7 macrophage cell line. - 4T1 murine breast cancer model with Balb/c background. | [50] |
| Hollow MnO_2_ (HMnO_2_ ) catalytic nanosystem camouflaged with Red blood cell membrane (mRBC)  [PMLR NPs] | Embedded with lactate oxidase (LOX) and a glycolysis inhibitor (PMLR).  Spherical and core-shell structure; 220-230nm (approx.) | Lactate oxidase (LOX) and glycolysis inhibitor (PMLR). | Tumor site | - Longer circulation time. - Accumulation at the tumor site. - Consumption of lactic acid in the TME by catalyzing its oxidation reaction via LOX. - Glycolysis inhibitor eliminates the source of lactic acid and blocks the adenosine triphosphate (ATP) supply. - Tumor growth inhibition. | - B16F10 cell line - Raw 267.4 cell line - B16F10tumor-bearing mice | [51] |
| Red blood cell (RBC) and platelet membrane-coated gold nanostars containing curcumin  [R/P-cGNS] | Star-shaped NPs; 162.1 ± 3.0 nm (approx.) | Curcumin | Macrophages  (via endosome/lysosome) | - Ability to target melanoma cells. - Display a controlled-release profile. - Inhibition of macrophage activation to balance the inflammatory responses. - Evasion of clearance of NPs by phagocytosis. - Anticancer and anti-inflammatory effects. - Combination of photothermal and chemotherapeutic effects. | Murine macrophage cell line  (RAW264.7) and melanoma cancer cell line B16-BL6 | [52] |
| Curcumin loaded serratiopeptidase nanoparticles  [Cur-SPD NPs] | Spherical; 175 ± 15 nm (approx.) | Curcumin and serratiopeptidase | Human macrophages | - Tumor suppression - Induced apoptosis - Inhibited migration of cancer cells - Stimulated ROS production in MCF-7 and HeLa cells. - Reduced IL-6 levels and increased TNFα level in THP1 cells. | MCF-7 (human breast cancer), THP1(human macrophages), and HeLa (human cervical cancer) cell lines | [53] |
| Poly (oligo (ethylene glycol) methacrylate) (POEG) carrier incorporated with indoximod (IND) for delivery of doxorubicin.  [POEG-b-PVBIND NPs] | A dual-functional polymeric carrier modified with pendent IND for co-delivery of  Doxorubicin (Dox).  Circular Micelles; 50.83 ± 1.25 nm (approx.) | Indoximod (IND) and  Doxorubicin (Dox) | Tumor site | - Sustained release of IND. - Promotion of tumor infiltration of CD8^+^ T cells. - Increased IFN-γ-production by CD8^+^ T cells. - Inhibition of immunosuppressive T regulatory cells (Tregs) | - 4T1.2 (murine breast cancer cell line) - Female BALB/c mice. - Orthotopic syngeneic murine breast cancer model (4T1.2) | [54] |
| Lipid-coated calcium phosphate (LCP) nanoparticle  [LCP-GMP] | Phosphorylated Gemcitabine drug loaded in calcium phosphate nano-scaffold encapsulated within lipid bilayer grafted with PEG chains.  Spherical core-shell nanostructure; ~30 nm  Surface molecules; PEG chains | Gemcitabine monophosphate (GMP) | Myeloid cells | - Induced apoptosis and decreased immunosuppressive effects within the tumor microenvironment (TME). - Reduced population of myeloid-derived suppressor cells (MDSCs) and regulatory T cells (Tregs). - Promoted macrophage polarization towards the M1 phenotype. - Enhanced CD8^+^ T-cell mediated immune responses. - Displayed tumor growth inhibition. | - C57BL/6 mice - Syngeneic mouse model of B16F10 melanoma | [55] |
| Polydopamine coated aluminum oxide (Al_2_O_3_) nanocarriers.  [pD-Al_2_O_3_] | Spherical core-shell nanostructure; 286.3 ± 4.2 nm to 344.4 ± 5.5 nm (approx.) | Cytosine-phosphate-guanine (CpG) | Cancer cells | - Aluminum oxide and CpG acts as an adjuvant to trigger cell-mediated immune responses. - Displayed high photothermal efficiency. - Released tumor-associated antigens due to tumor cell death leading to dendritic cell maturation. - Combined therapy aided the elimination of residual tumor cells and reduced the risk of tumor recurrence. | - B16F10 melanoma cell line - C57BL/6mice - B16F10 melanoma allografts in mice. | [56] |

**References**

1. Zanganeh S, Hutter G, Spitler R, Lenkov O, Mahmoudi M, Shaw A, Pajarinen JS, Nejadnik H, Goodman S, Moseley M, Coussens LM, Daldrup-Link HE. Iron oxide nanoparticles inhibit tumour growth by inducing pro-inflammatory macrophage polarization in tumour tissues. Nat Nanotechnol. 2016 Nov;11(11):986-994. doi: 10.1038/nnano.2016.168. Epub 2016 Sep 26. PMID: 27668795; PMCID: PMC5198777.
2. Mulens-Arias V, Rojas JM, Pérez-Yagüe S, Morales MP, Barber DF. Polyethylenimine-coated SPIONs trigger macrophage activation through TLR-4 signaling and ROS production and modulate podosome dynamics. Biomaterials. 2015 Jun;52:494-506. doi: 10.1016/j.biomaterials.2015.02.068. Epub 2015 Mar 18. PMID: 25818455.
3. Tang J, Chen Z, Sun B, Dong J, Liu J, Zhou H, Wang L, Bai R, Miao Q, Zhao Y, Chen C, Liu Y. Polyhydroxylated fullerenols regulate macrophage for cancer adoptive immunotherapy and greatly inhibit the tumor metastasis. Nanomedicine. 2016 May;12(4):945-954. doi: 10.1016/j.nano.2015.11.021. Epub 2015 Dec 28. PMID: 26733256.
4. Meng H, Xing G, Blanco E, Song Y, Zhao L, Sun B, Li X, Wang PC, Korotcov A, Li W, Liang XJ, Chen C, Yuan H, Zhao F, Chen Z, Sun T, Chai Z, Ferrari M, Zhao Y. Gadolinium metallofullerenol nanoparticles inhibit cancer metastasis through matrix metalloproteinase inhibition: imprisoning instead of poisoning cancer cells. Nanomedicine. 2012 Feb;8(2):136-46. doi: 10.1016/j.nano.2011.08.019. Epub 2011 Sep 17. PMID: 21930111; PMCID: PMC3510664.
5. Zhang F, Li F, Lu GH, Nie W, Zhang L, Lv Y, Bao W, Gao X, Wei W, Pu K, Xie HY. Engineering Magnetosomes for Ferroptosis/Immunomodulation Synergism in Cancer. ACS Nano. 2019 May 28;13(5):5662-5673. doi: 10.1021/acsnano.9b00892. Epub 2019 May 6. PMID: 31046234.
6. Fatima F, Bajpai P, Pathak N, Singh S, Priya S, Verma SR. Antimicrobial and immunomodulatory efficacy of extracellularly synthesized silver and gold nanoparticles by a novel phosphate solubilizing fungus Bipolaris tetramera. BMC Microbiol. 2015 Feb 27;15:52. doi: 10.1186/s12866-015-0391-y. PMID: 25881309; PMCID: PMC4364495.
7. Min Y, Roche KC, Tian S, Eblan MJ, McKinnon KP, Caster JM, Chai S, Herring LE, Zhang L, Zhang T, DeSimone JM, Tepper JE, Vincent BG, Serody JS, Wang AZ. Antigen-capturing nanoparticles improve the abscopal effect and cancer immunotherapy. Nat Nanotechnol. 2017 Sep;12(9):877-882. doi: 10.1038/nnano.2017.113. Epub 2017 Jun 26. PMID: 28650437; PMCID: PMC5587366.
8. Choo YW, Kang M, Kim HY, Han J, Kang S, Lee JR, Jeong GJ, Kwon SP, Song SY, Go S, Jung M, Hong J, Kim BS. M1 Macrophage-Derived Nanovesicles Potentiate the Anticancer Efficacy of Immune Checkpoint Inhibitors. ACS Nano. 2018 Sep 25;12(9):8977-8993. doi: 10.1021/acsnano.8b02446. Epub 2018 Aug 29. PMID: 30133260.
9. Fuchs AK, Syrovets T, Haas KA, Loos C, Musyanovych A, Mailänder V, Landfester K, Simmet T. Carboxyl- and amino-functionalized polystyrene nanoparticles differentially affect the polarization profile of M1 and M2 macrophage subsets. Biomaterials. 2016 Apr;85:78-87. doi: 10.1016/j.biomaterials.2016.01.064. Epub 2016 Jan 29. PMID: 26854393.
10. Kodali V, Littke MH, Tilton SC, Teeguarden JG, Shi L, Frevert CW, Wang W, Pounds JG, Thrall BD. Dysregulation of macrophage activation profiles by engineered nanoparticles. ACS Nano. 2013 Aug 27;7(8):6997-7010. doi: 10.1021/nn402145t. Epub 2013 Jul 9. PMID: 23808590; PMCID: PMC3756554.
11. Laskar A, Eilertsen J, Li W, Yuan XM. SPION primes THP1 derived M2 macrophages towards M1-like macrophages. Biochem Biophys Res Commun. 2013 Nov 29;441(4):737-42. doi: 10.1016/j.bbrc.2013.10.115. Epub 2013 Oct 30. PMID: 24184477.
12. Zhang W, Cao S, Liang S, Tan CH, Luo B, Xu X, Saw PE. Differently Charged Super-Paramagnetic Iron Oxide Nanoparticles Preferentially Induced M1-Like Phenotype of Macrophages. Front Bioeng Biotechnol. 2020 May 29;8:537. doi: 10.3389/fbioe.2020.00537. PMID: 32548111; PMCID: PMC7272720.
13. Liu X, Yan B, Li Y, Ma X, Jiao W, Shi K, Zhang T, Chen S, He Y, Liang XJ, Fan H. Graphene Oxide-Grafted Magnetic Nanorings Mediated Magnetothermodynamic Therapy Favoring Reactive Oxygen Species-Related Immune Response for Enhanced Antitumor Efficacy. ACS Nano. 2020 Feb 25;14(2):1936-1950. doi: 10.1021/acsnano.9b08320. Epub 2020 Jan 24. PMID: 31961656.
14. Kinaret PAS, Scala G, Federico A, Sund J, Greco D. Carbon Nanomaterials Promote M1/M2 Macrophage Activation. Small. 2020 May;16(21):e1907609. doi: 10.1002/smll.201907609. Epub 2020 Apr 6. PMID: 32250056.
15. Plebanek MP, Bhaumik D, Bryce PJ, Thaxton CS. Scavenger Receptor Type B1 and Lipoprotein Nanoparticle Inhibit Myeloid-Derived Suppressor Cells. Mol Cancer Ther. 2018 Mar;17(3):686-697. doi: 10.1158/1535-7163.MCT-17-0981. Epub 2017 Dec 27. PMID: 29282300; PMCID: PMC5935575.
16. Li CX, Zhang Y, Dong X, Zhang L, Liu MD, Li B, Zhang MK, Feng J, Zhang XZ. Artificially Reprogrammed Macrophages as Tumor-Tropic Immunosuppression-Resistant Biologics to Realize Therapeutics Production and Immune Activation. Adv Mater. 2019 Apr;31(15):e1807211. doi: 10.1002/adma.201807211. Epub 2019 Feb 25. PMID: 30803083.
17. Zhang YN, Lazarovits J, Poon W, Ouyang B, Nguyen LNM, Kingston BR, Chan WCW. Nanoparticle Size Influences Antigen Retention and Presentation in Lymph Node Follicles for Humoral Immunity. Nano Lett. 2019 Oct 9;19(10):7226-7235. doi: 10.1021/acs.nanolett.9b02834. Epub 2019 Sep 17. PMID: 31508968.
18. Deng RH, Zou MZ, Zheng D, Peng SY, Liu W, Bai XF, Chen HS, Sun Y, Zhou PH, Zhang XZ. Nanoparticles from Cuttlefish Ink Inhibit Tumor Growth by Synergizing Immunotherapy and Photothermal Therapy. ACS Nano. 2019 Aug 27;13(8):8618-8629. doi: 10.1021/acsnano.9b02993. Epub 2019 Jul 1. PMID: 31246413.
19. Zhang Y , Sha W , Zhang X , Cheng M , Wu Q , Wang W , Yuan Z . Zwitterionic chitooligosaccharide-modified ink-blue titanium dioxide nanoparticles with inherent immune activation for enhanced photothermal therapy. Biomater Sci. 2019 Nov 19;7(12):5027-5034. doi: 10.1039/c9bm01170f. PMID: 31528908.
20. Jiang Q, Luo Z, Men Y, Yang P, Peng H, Guo R, Tian Y, Pang Z, Yang W. Red blood cell membrane-camouflaged melanin nanoparticles for enhanced photothermal therapy. Biomaterials. 2017 Oct;143:29-45. doi: 10.1016/j.biomaterials.2017.07.027. Epub 2017 Jul 20. PMID: 28756194.
21. Chang M, Hou Z, Wang M, Wang M, Dang P, Liu J, Shu M, Ding B, Al Kheraif AA, Li C, Lin J. Cu2 MoS4 /Au Heterostructures with Enhanced Catalase-Like Activity and Photoconversion Efficiency for Primary/Metastatic Tumors Eradication by Phototherapy-Induced Immunotherapy. Small. 2020 Apr;16(14):e1907146. doi: 10.1002/smll.201907146. Epub 2020 Mar 12. PMID: 32162784.
22. Korangath P, Barnett JD, Sharma A, Henderson ET, Stewart J, Yu SH, Kandala SK, Yang CT, Caserto JS, Hedayati M, Armstrong TD, Jaffee E, Gruettner C, Zhou XC, Fu W, Hu C, Sukumar S, Simons BW, Ivkov R. Nanoparticle interactions with immune cells dominate tumor retention and induce T cell-mediated tumor suppression in models of breast cancer. Sci Adv. 2020 Mar 25;6(13):eaay1601. doi: 10.1126/sciadv.aay1601. PMID: 32232146; PMCID: PMC7096167.
23. Luo L, Iqbal MZ, Liu C, Xing J, Akakuru OU, Fang Q, Li Z, Dai Y, Li A, Guan Y, Wu A. Engineered nano-immunopotentiators efficiently promote cancer immunotherapy for inhibiting and preventing lung metastasis of melanoma. Biomaterials. 2019 Dec;223:119464. doi: 10.1016/j.biomaterials.2019.119464. Epub 2019 Sep 3. PMID: 31525691.
24. Zhang D, Wu T, Qin X, Qiao Q, Shang L, Song Q, Yang C, Zhang Z. Intracellularly Generated Immunological Gold Nanoparticles for Combinatorial Photothermal Therapy and Immunotherapy against Tumor. Nano Lett. 2019 Sep 11;19(9):6635-6646. doi: 10.1021/acs.nanolett.9b02903. Epub 2019 Aug 12. PMID: 31393134.
25. Liu Y, Han Y, Dong H, Wei X, Shi D, Li Y. Ca2+-Mediated Surface Polydopamine Engineering to Program Dendritic Cell Maturation. ACS Appl Mater Interfaces. 2020 Jan 22;12(3):4163-4173. doi: 10.1021/acsami.9b20997. Epub 2020 Jan 10. PMID: 31891476.
26. Ma W, Chen M, Kaushal S, McElroy M, Zhang Y, Ozkan C, Bouvet M, Kruse C, Grotjahn D, Ichim T, Minev B. PLGA nanoparticle-mediated delivery of tumor antigenic peptides elicits effective immune responses. Int J Nanomedicine. 2012;7:1475-87. doi: 10.2147/IJN.S29506. Epub 2012 Mar 15. PMID: 22619507; PMCID: PMC3356185.
27. Kim H, Niu L, Larson P, Kucaba TA, Murphy KA, James BR, Ferguson DM, Griffith TS, Panyam J. Polymeric nanoparticles encapsulating novel TLR7/8 agonists as immunostimulatory adjuvants for enhanced cancer immunotherapy. Biomaterials. 2018 May;164:38-53. doi: 10.1016/j.biomaterials.2018.02.034. Epub 2018 Feb 17. PMID: 29482062.
28. Han HD, Byeon Y, Kang TH, Jung ID, Lee JW, Shin BC, Lee YJ, Sood AK, Park YM. Toll-like receptor 3-induced immune response by poly(d,l-lactide-co-glycolide) nanoparticles for dendritic cell-based cancer immunotherapy. Int J Nanomedicine. 2016 Nov 2;11:5729-5742. doi: 10.2147/IJN.S109001. PMID: 27843314; PMCID: PMC5098754.
29. Fang RH, Hu CM, Luk BT, Gao W, Copp JA, Tai Y, O'Connor DE, Zhang L. Cancer cell membrane-coated nanoparticles for anticancer vaccination and drug delivery. Nano Lett. 2014;14(4):2181-8. doi: 10.1021/nl500618u. Epub 2014 Mar 28. PMID: 24673373; PMCID: PMC3985711.
30. Yang R, Xu J, Xu L, Sun X, Chen Q, Zhao Y, Peng R, Liu Z. Cancer Cell Membrane-Coated Adjuvant Nanoparticles with Mannose Modification for Effective Anticancer Vaccination. ACS Nano. 2018 Jun 26;12(6):5121-5129. doi: 10.1021/acsnano.7b09041. Epub 2018 May 22. PMID: 29771487.
31. Guo Y, Wang D, Song Q, Wu T, Zhuang X, Bao Y, Kong M, Qi Y, Tan S, Zhang Z. Erythrocyte Membrane-Enveloped Polymeric Nanoparticles as Nanovaccine for Induction of Antitumor Immunity against Melanoma. ACS Nano. 2015 Jul 28;9(7):6918-33. doi: 10.1021/acsnano.5b01042. Epub 2015 Jul 14. PMID: 26153897.
32. Zhang Z, Tongchusak S, Mizukami Y, Kang YJ, Ioji T, Touma M, Reinhold B, Keskin DB, Reinherz EL, Sasada T. Induction of anti-tumor cytotoxic T cell responses through PLGA-nanoparticle mediated antigen delivery. Biomaterials. 2011 May;32(14):3666-78. doi: 10.1016/j.biomaterials.2011.01.067. Epub 2011 Feb 23. PMID: 21345488.
33. Yaman S, Ramachandramoorthy H, Oter G, Zhukova D, Nguyen T, Sabnani MK, Weidanz JA, Nguyen KT. Melanoma Peptide MHC Specific TCR Expressing T-Cell Membrane Camouflaged PLGA Nanoparticles for Treatment of Melanoma Skin Cancer. Front Bioeng Biotechnol. 2020 Aug 11;8:943. doi: 10.3389/fbioe.2020.00943. PMID: 32850765; PMCID: PMC7431670.
34. Song C, Noh YW, Lim YT. Polymer nanoparticles for cross-presentation of exogenous antigens and enhanced cytotoxic T-lymphocyte immune response. Int J Nanomedicine. 2016 Aug 5;11:3753-64. doi: 10.2147/IJN.S110796. PMID: 27540289; PMCID: PMC4981168.
35. Uto T, Akagi T, Yoshinaga K, Toyama M, Akashi M, Baba M. The induction of innate and adaptive immunity by biodegradable poly(γ-glutamic acid) nanoparticles via a TLR4 and MyD88 signaling pathway. Biomaterials. 2011 Aug;32(22):5206-12. doi: 10.1016/j.biomaterials.2011.03.052. Epub 2011 Apr 13. PMID: 21492934.
36. Ahmed KK, Geary SM, Salem AK. Development and Evaluation of Biodegradable Particles Coloaded With Antigen and the Toll-Like Receptor Agonist, Pentaerythritol Lipid A, as a Cancer Vaccine. J Pharm Sci. 2016 Mar;105(3):1173-9. doi: 10.1016/j.xphs.2015.11.042. Epub 2016 Jan 30. PMID: 26886334; PMCID: PMC4775392.
37. Zhou Q, Zhang Y, Du J, Li Y, Zhou Y, Fu Q, Zhang J, Wang X, Zhan L. Different-Sized Gold Nanoparticle Activator/Antigen Increases Dendritic Cells Accumulation in Liver-Draining Lymph Nodes and CD8+ T Cell Responses. ACS Nano. 2016 Feb 23;10(2):2678-92. doi: 10.1021/acsnano.5b07716. Epub 2016 Jan 15. PMID: 26771692.
38. Wang B, Van Herck S, Chen Y, Bai X, Zhong Z, Deswarte K, Lambrecht BN, Sanders NN, Lienenklaus S, Scheeren HW, David SA, Kiessling F, Lammers T, De Geest BG, Shi Y. Potent and Prolonged Innate Immune Activation by Enzyme-Responsive Imidazoquinoline TLR7/8 Agonist Prodrug Vesicles. J Am Chem Soc. 2020 Jul 15;142(28):12133-12139. doi: 10.1021/jacs.0c01928. Epub 2020 Jun 30. PMID: 32524819; PMCID: PMC7116109.
39. Belhadj Z, He B, Deng H, Song S, Zhang H, Wang X, Dai W, Zhang Q. A combined "eat me/don't eat me" strategy based on extracellular vesicles for anticancer nanomedicine. J Extracell Vesicles. 2020 Aug 19;9(1):1806444. doi: 10.1080/20013078.2020.1806444. PMID: 32944191; PMCID: PMC7480498.
40. Darling R, Senapati S, Christiansen J, Liu L, Ramer-Tait AE, Narasimhan B, Wannemuehler M. Polyanhydride Nanoparticles Induce Low Inflammatory Dendritic Cell Activation Resulting in CD8+ T Cell Memory and Delayed Tumor Progression. Int J Nanomedicine. 2020 Sep 7;15:6579-6592. doi: 10.2147/IJN.S261041. PMID: 32982219; PMCID: PMC7490050.
41. Wilson DR, Sen R, Sunshine JC, Pardoll DM, Green JJ, Kim YJ. Biodegradable STING agonist nanoparticles for enhanced cancer immunotherapy. Nanomedicine. 2018 Feb;14(2):237-246. doi: 10.1016/j.nano.2017.10.013. Epub 2017 Nov 7. PMID: 29127039; PMCID: PMC6035751.
42. Tao Y, Ju E, Ren J, Qu X. Immunostimulatory oligonucleotides-loaded cationic graphene oxide with photothermally enhanced immunogenicity for photothermal/immune cancer therapy. Biomaterials. 2014 Dec;35(37):9963-9971. doi: 10.1016/j.biomaterials.2014.08.036. Epub 2014 Sep 15. PMID: 25224368.
43. Shevtsov MA, Nikolaev BP, Yakovleva LY, Parr MA, Marchenko YY, Eliseev I, Yudenko A, Dobrodumov AV, Zlobina O, Zhakhov A, Ischenko AM, Pitkin E, Multhoff G. 70-kDa heat shock protein coated magnetic nanocarriers as a nanovaccine for induction of anti-tumor immune response in experimental glioma. J Control Release. 2015 Dec 28;220(Pt A):329-340. doi: 10.1016/j.jconrel.2015.10.051. Epub 2015 Oct 29. PMID: 26522072.
44. Barati N, Nikpoor AR, Razazan A, Mosaffa F, Badiee A, Arab A, Gholizadeh Z, Behravan J, Jaafari MR. Nanoliposomes carrying HER2/neu-derived peptide AE36 with CpG-ODN exhibit therapeutic and prophylactic activities in a mice TUBO model of breast cancer. Immunol Lett. 2017 Oct;190:108-117. doi: 10.1016/j.imlet.2017.07.009. Epub 2017 Jul 21. PMID: 28736158.
45. Rueda F, Eich C, Cordobilla B, Domingo P, Acosta G, Albericio F, Cruz LJ, Domingo JC. Effect of TLR ligands co-encapsulated with multiepitopic antigen in nanoliposomes targeted to human DCs via Fc receptor for cancer vaccines. Immunobiology. 2017 Nov;222(11):989-997. doi: 10.1016/j.imbio.2017.06.002. Epub 2017 Jun 10. PMID: 28624137.
46. Kapadia CH, Tian S, Perry JL, Sailer D, Christopher Luft J, DeSimone JM. Extending antigen release from particulate vaccines results in enhanced antitumor immune response. J Control Release. 2018 Jan 10;269:393-404. doi: 10.1016/j.jconrel.2017.11.020. Epub 2017 Nov 13. PMID: 29146244.
47. Zhou H, Fan Z, Deng J, Lemons PK, Arhontoulis DC, Bowne WB, Cheng H. Hyaluronidase Embedded in Nanocarrier PEG Shell for Enhanced Tumor Penetration and Highly Efficient Antitumor Efficacy. Nano Lett. 2016 May 11;16(5):3268-77. doi: 10.1021/acs.nanolett.6b00820. Epub 2016 Apr 8. PMID: 27057591.
48. Cheng N, Watkins-Schulz R, Junkins RD, David CN, Johnson BM, Montgomery SA, Peine KJ, Darr DB, Yuan H, McKinnon KP, Liu Q, Miao L, Huang L, Bachelder EM, Ainslie KM, Ting JP. A nanoparticle-incorporated STING activator enhances antitumor immunity in PD-L1-insensitive models of triple-negative breast cancer. JCI Insight. 2018 Nov 15;3(22):e120638. doi: 10.1172/jci.insight.120638. PMID: 30429378; PMCID: PMC6302949.
49. Kim SY, Kim S, Kim JE, Lee SN, Shin IW, Shin HS, Jin SM, Noh YW, Kang YJ, Kim YS, Kang TH, Park YM, Lim YT. Lyophilizable and Multifaceted Toll-like Receptor 7/8 Agonist-Loaded Nanoemulsion for the Reprogramming of Tumor Microenvironments and Enhanced Cancer Immunotherapy. ACS Nano. 2019 Nov 26;13(11):12671-12686. doi: 10.1021/acsnano.9b04207. Epub 2019 Oct 11. PMID: 31589013.
50. Song M, Liu T, Shi C, Zhang X, Chen X. Bioconjugated Manganese Dioxide Nanoparticles Enhance Chemotherapy Response by Priming Tumor-Associated Macrophages toward M1-like Phenotype and Attenuating Tumor Hypoxia. ACS Nano. 2016 Jan 26;10(1):633-647. doi: 10.1021/acsnano.5b06779. Epub 2015 Dec 14. Erratum in: ACS Nano. 2016 Mar 22;10(3):3872. Zhang, Xiangzhong [corrected to Zhang, Xianzhong]. PMID: 26650065; PMCID: PMC5242343.
51. Gao F, Tang Y, Liu WL, Zou MZ, Huang C, Liu CJ, Zhang XZ. Intra/Extracellular Lactic Acid Exhaustion for Synergistic Metabolic Therapy and Immunotherapy of Tumors. Adv Mater. 2019 Dec;31(51):e1904639. doi: 10.1002/adma.201904639. Epub 2019 Nov 6. PMID: 31692128.
52. Kim MW, Lee G, Niidome T, Komohara Y, Lee R, Park YI. Platelet-Like Gold Nanostars for Cancer Therapy: The Ability to Treat Cancer and Evade Immune Reactions. Front Bioeng Biotechnol. 2020 Feb 25;8:133. doi: 10.3389/fbioe.2020.00133. PMID: 32158752; PMCID: PMC7051916.
53. Jaiswal S, Mishra P. Co-delivery of curcumin and serratiopeptidase in HeLa and MCF-7 cells through nanoparticles show improved anti-cancer activity. Mater Sci Eng C Mater Biol Appl. 2018 Nov 1;92:673-684. doi: 10.1016/j.msec.2018.07.025. Epub 2018 Jul 11. PMID: 30184794.
54. Wan Z, Sun J, Xu J, Moharil P, Chen J, Xu J, Zhu J, Li J, Huang Y, Xu P, Ma X, Xie W, Lu B, Li S. Dual functional immunostimulatory polymeric prodrug carrier with pendent indoximod for enhanced cancer immunochemotherapy. Acta Biomater. 2019 May;90:300-313. doi: 10.1016/j.actbio.2019.03.048. Epub 2019 Mar 28. PMID: 30930305; PMCID: PMC6513707.
55. Zhang Y, Bush X, Yan B, Chen JA. Gemcitabine nanoparticles promote antitumor immunity against melanoma. Biomaterials. 2019 Jan;189:48-59. doi: 10.1016/j.biomaterials.2018.10.022. Epub 2018 Oct 20. PMID: 30388589; PMCID: PMC6281175.
56. Chen W, Qin M, Chen X, Wang Q, Zhang Z, Sun X. Combining photothermal therapy and immunotherapy against melanoma by polydopamine-coated Al2O3 nanoparticles. Theranostics. 2018 Mar 8;8(8):2229-2241. doi: 10.7150/thno.24073. PMID: 29721075; PMCID: PMC5928883.

**
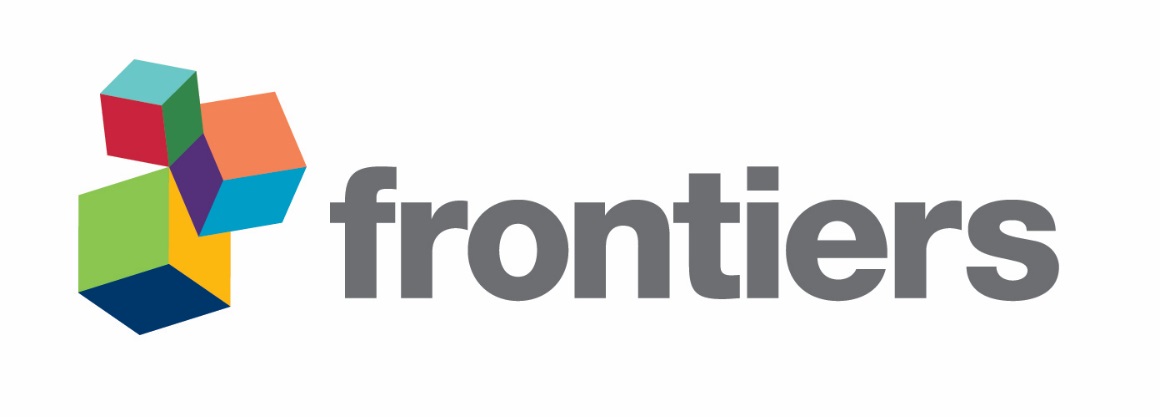
**
